# Microbial and bryospheric photosynthesis of boreal peatlands have peatland‐type‐specific responses to long‐term drying

**DOI:** 10.1111/nph.70519

**Published:** 2025-09-02

**Authors:** Olivia Kuuri‐Riutta, Marie Le Geay, Vincent E. J. Jassey, Janna M. Barel, Anna M. Laine, Henni Ylänne, Eeva‐Stiina Tuittila

**Affiliations:** ^1^ School of Forest Sciences University of Eastern Finland 80101 Joensuu Finland; ^2^ Centre de Recherche sur la Biodiversité et l'Environnement (CRBE) Université de Toulouse, CNRS, IRD, Toulouse INP 31062 Toulouse France; ^3^ Ecology and Biodiversity, Department of Biology, Institute of Environmental Biology Utrecht University PO Box 80125 Utrecht 3584 TC the Netherlands

**Keywords:** Cyanobacteria, microalgae, microbial photosynthesis, moss microbiome, peatland drying, *Sphagnum*

## Abstract

- The impacts of drying on bryospheric photosynthesis are poorly understood. Utilising a 20‐yr‐long experiment, we quantified the effects of long‐term water level drawdown (WLD) on links between bryospheric photosynthesis, microbial community composition, decomposition, and environmental variables.
- The community structure of photoautotrophic microbes was investigated using metabarcoding and quantitative polymerase chain reaction. Microbial photosynthesis was measured as photosynthesis efficiency (φPSII) and maximum electron transport rate through photosystem II (ETR_max_). Bryospheric photosynthesis was measured using an infrared gas flux analyser.
- Chlorophyta, Cyanobacteria, and Ochrophyta were dominant photoautotrophic phyla in all study areas, but communities had site‐specific WLD responses. WLD increased microbial and bryospheric photosynthesis in the poor fen. ETR_max_ was promoted by soil phosphorus concentration, φPSII, and Chlorophyta abundance, and bryospheric photosynthesis by Cyanobacteria abundance, a deep water table, soil sulphur concentration, and decomposition. In undrained areas, high abundance of Cyanobacteria promoted soil nitrogen concentration and, therefore, photosynthesis. In WLD areas, these connections were lost, but bryospheric photosynthesis benefited from WLD directly.
- Our study confirms that photoautotrophic microbes, especially Cyanobacteria and Chlorophyta, are important contributors to bryospheric photosynthesis in pristine boreal peatlands. Furthermore, increased bryospheric photosynthesis after drying may offset carbon loss from increased decomposition, but this depends on the site characteristics.

## Introduction

Peatlands are a major player in climate regulation: They currently store 30% of global terrestrial carbon (Gorham, 1991; Nichols & Peteet, 2019). A substantial proportion of carbon sequestration in peatlands is conducted by peat mosses (*Sphagnum* spp., Kokkonen *et al*., 2022), whose functioning is highly affected by photoautotrophic microbial communities that fix both carbon and nitrogen from the atmosphere (Larmola *et al*., 2014; Kostka *et al*., 2016; Hamard *et al*., 2021a; Jassey *et al*., 2022a). As mosses and microbes live in such a close association, we refer to them in their entirety by the term bryosphere, which originally covers also soil fauna (Lindo & Gonzalez, 2010). Accordingly, photoautotrophic microbes associated with mosses have been estimated to account for 4–30% of the bryospheric photosynthesis and significantly promote moss biomass increment through nitrogen provision (Berg *et al*., 2013; Larmola *et al*., 2014; Hamard *et al*., 2021a; Jassey *et al*., 2022b). Yet, we have little understanding of how the ongoing climate change affects the various photoautotrophic microbes in peatlands (Kilner *et al*., 2024; Hamard *et al*., 2025), and how these microbial responses feed back to bryospheric photosynthesis.

Ongoing climatic and anthropogenic changes are suggested to lead to widespread drying of boreal peatlands, seen as water table (WT) decline and reduced moisture availability in the peat moss layer (Dimitrov *et al*., 2011; Gong *et al*., 2012; Helbig *et al*., 2020). If the site fertility is not limiting, drying typically promotes the establishment of shrubs and trees at the cost of mire vegetation, such as peat mosses (Mäkiranta *et al*., 2018; Kokkonen *et al*., 2019b, 2022). This, in turn, affects litter quality and quantity, intensifies shading to the moss layer, decreases soil temperature, lowers soil pH (Heikurainen & Seppälä, 1963; Minkkinen *et al*., 1999; Briones *et al*., 2022), and increases soil nutrient concentrations (Straková *et al*., 2012; Munir *et al*., 2017). Cumulatively, these changes have been found to reduce the cover and diversity of peat mosses and either reduce or increase their biomass, but to have little impact on their long‐term photosynthetic capacity (Hájek *et al*., 2009; Kangas *et al*., 2014; Kokkonen *et al*., 2019b, 2022). The surprising resilience of bryospheric photosynthesis has been suggested to be driven by trait plasticity within mosses (Jassey & Signarbieux, 2019). Yet, a part of it could be mediated by changes in the activity and composition of moss‐associated microbial communities that affect bryospheric photosynthesis via signal chemicals (Carrell *et al*., 2022a), nitrogen provision (Larmola *et al*., 2014), and by compensating for the reduced photosynthetic activity in mosses by fixing atmospheric carbon (Hamard *et al*., 2021a). In fact, plant‐associated microbiota has been suggested as an extension of the plant phenotype, as its role in the nitrogen acquisition of plants is crucial (Cheng *et al*., 2024). Especially, nitrogen‐fixing Cyanobacteria are considered important symbionts for *Sphagnum* mosses (Carrell *et al*., 2022b; Álvarez *et al*., 2023). Thus, microbes could have an overlooked role in regulating bryospheric photosynthesis and its response to environmental changes.

The existing knowledge regarding the responses of moss‐associated photoautotrophic microbes to peatland drying remains somewhat controversial. Photoautotrophic microbes have been reported to suffer from both drying (Briones *et al*., 2022; Jassey *et al*., 2022a) and intensified shading (Payne *et al*., 2016; Lamentowicz *et al*., 2020), leading to lower net CO_2_ fixation rates in drier conditions (Jassey *et al*., 2022a). However, peatland drying has also been projected to accelerate the metabolic activity of photoautotrophic microbial communities (Le Geay *et al*., 2024a), and microbial photosynthesis has been shown to peak in dry and shaded microhabitats due to relatively high microbial biomass, Chl*a* concentration, electron transport rate, and shade adaptation in the light‐harvesting machinery (Perrine *et al*., 2012; Hamard *et al*., 2021b). In addition, soil nutrient concentrations are often relatively high in drying conditions due to the accelerated mineralisation (Laiho, 2006) and, possibly, nitrogen fixation of dry‐tolerant Cyanobacteria (Nichols & Adams, 1982 as cited in Carey *et al*., 2012, Osborne & Raven, 1986). The availability of nitrogen and phosphorus regulates microbial photosynthesis in peatlands (Wyatt & Turetsky, 2015; DeColibus *et al*., 2017), and therefore, this increased nutrient availability can promote the growth and productivity of photoautotrophic microbes and/or *Sphagnum* mosses in drying conditions (Hamard *et al*., 2025). Thus, the increased contribution of microbes to bryospheric carbon fixation, along with additional nitrogen provision from Cyanobacteria and increased nutrient mineralisation, could potentially explain the drying resistance of bryospheric photosynthesis. Due to the multiple concurrent changes, the overall impact of climate‐induced drying on the activity, community composition, and diversity of photoautotrophic microbes remains to be assessed. Such predictions are further complicated by the fact that, as with the responses of vascular vegetation (Kokkonen *et al*., 2019b, 2022), microbial responses to long‐term drying could be dictated by the site fertility level (Urbanová & Bárta, 2016).

This study aimed to understand how long‐term drying affects microbial and bryospheric carbon fixation through various changes in ecosystems. We further aim to address how peatland types alter these relationships. To do so, we utilised a 20‐yr‐old water level drawdown (WLD) experiment that covers three different peatland types typical for the boreal zone, namely rich fen, poor fen, and bog. We hypothesise (H1) WLD to alter the moss‐associated photoautotrophic microbial community, favouring especially Cyanobacteria, and (H2) to promote the photosynthetic capacity of both microbes and the bryosphere as a whole. We further hypothesise (H3) these changes to follow the same pattern as previously described in the composition and photosynthesis of vegetation (Kokkonen *et al*., 2019b, 2022; Laine *et al*., 2021), that is the most pronounced impact in the rich fen and the weakest in the bog. In addition, we aim to investigate the drivers of microbial and bryospheric photosynthesis in general, and specifically test how nitrogen provided by Cyanobacteria and decomposition promote photosynthesis in pristine and drying conditions.

## Materials and Methods

### Study site

**Fig. 1:**
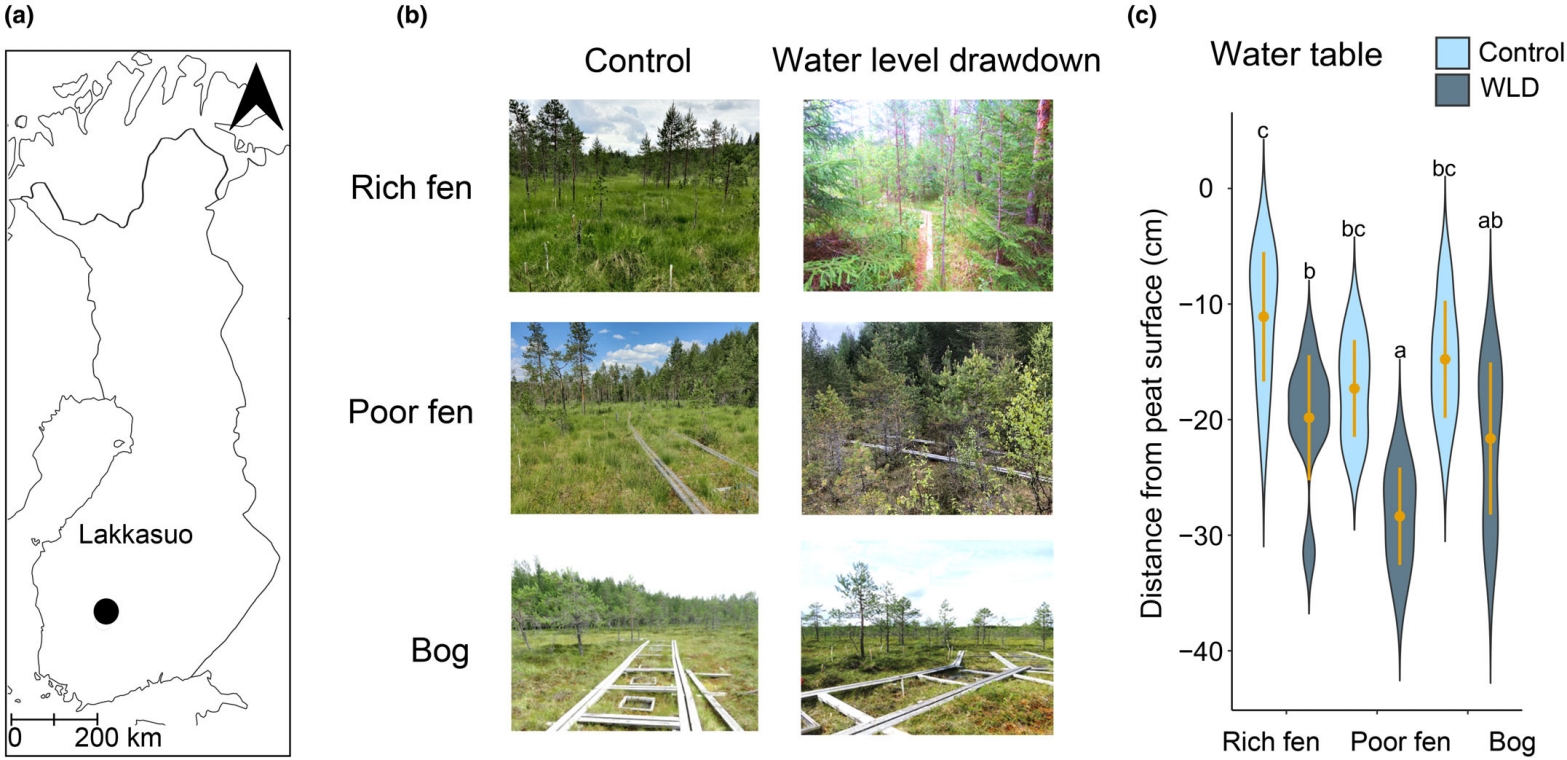
Description of the study site. (a) The location of the study site, Lakkasuo peatland. (b) Photographs of the six study areas. (c) Differences in water table level in the Lakkasuo study areas, measured four times from permanent water wells in June–August 2022. The violins illustrate the distribution of the data and the kernel probability density of the data at different values. The yellow dots indicate mean values, and the whiskers show SD. Letters indicate statistically significant differences according to Tukey's pairwise comparison (*P*‐value < 0.05).

Our study site is located in an eccentric raised peatland complex, Lakkasuo, located in Southern Finland (61°47′N, 24°18′E; Fig. 1a). The mean annual temperature in the area is 3.5°C, and mean annual precipitation is 711 mm (*ICOS Ecosystem Station Labelling Report*, *Station*: *FI‐Hyy (Hyytiälä*), 2019). We studied the effects of long‐term WLD using a field experiment that was established in 2000–2001 to simulate climate‐induced drying (Laine *et al*., 2021; Fig. 1b). The experiment consists of three study sites that have been divided into a control area and an experimental WLD area (six study areas altogether). Each study area (i.e. each treatment in each site) has 8–10 permanent sample plots: eight in both areas in the rich fen, nine in both areas in the poor fen and in the bog control area, and 10 in the bog WLD area, 53 altogether. All the data in this study have been collected from those sample plots or their immediate proximity.

The sites represent three different peatland types that differ in fertility: rich fen and poor fen, which receive water and minerals from surrounding mineral land, and a bog that receives water only from precipitation. The WLD areas are surrounded by 30‐cm‐deep ditches that deepened WT by 9 cm on average compared to the control areas in the study year 2022 (Fig. 1c). WLD has caused drainage succession in vegetation that differs between the sites: In the fen sites, the WLD areas have grown a young tree stand, while in the bog, the vegetation has changed only slightly (Kokkonen *et al*., 2019b; Fig. 1b).

### Field and laboratory measurements

#### Environmental variables and decomposition rate

We obtained information on the plot‐wise peat nutrient concentration and pH (0–10 cm depth) from previous field campaigns (measured in 2016; Kokkonen *et al*., 2019b) and complemented these with additional measurements of the water table level, shading intensity, moss moisture content, and soil temperature at 5 and 15 cm depths (Supporting Information Table S1) that were measured in 2022.

Decomposition was investigated with the Tea Bag Index, commonly used to quantify the decomposition potential of a site (Keuskamp *et al*., 2013; Sarneel *et al*., 2024). Tea provides a standardised substrate equally foreign for all study sites, which quantifies solely the effects of the decomposer community and abiotic factors on decomposition. The mass loss of rooibos and stabilisation factor based on the mass loss of green tea are subsequently used to estimate the site potential decomposition rate (*k*_TBI_). We followed the protocol described in Keuskamp *et al*. (2013). At the beginning of the growing season (June 2022), a bag of Lipton's green tea (EAN 8722700055525) and a bag of rooibos (EAN 8711327514348) were buried at an 8 cm depth next to each of the 53 permanent sample plots. The bags were retrieved after 3 months, and the remaining dry mass of the tea was determined.

#### Community composition of photoautotrophic microbes

For determining the composition of the photoautotrophic microbial community, we collected the living part of three to five individual moss shoots from each permanent sample plot in summer 2022. We collected the dominant moss species in each sample plot, which was *Sphagnum* spp. in all but two sample plots in the rich fen WLD area that were dominated by *Dicranum polysetum*. The plant material was immediately placed in 5‐ml Eppendorf tubes containing 4 ml of RNAlater to preserve the DNA before extractions until we came back to the laboratory. Total DNA was extracted with the DNeasy PowerSoil Pro Kit (Qiagen), following the manufacturer's instructions. Briefly, moss shoots were cut into small pieces, and up to 250 mg was added to the PowerBead Pro Tube before proceeding to the extraction steps. The lysis step was conducted on a TissueLyser II (Qiagen). Directly after elution, DNA was quantified on a Nanodrop ND‐1000 spectrophotometer and subjected to sequencing. To target oxygenic photoautotrophs (both eukaryotes and prokaryotes), we used the primer pair P23SrV_f1: 5′‐GGA CAG AAA GAC CCT ATG AA‐3′/P23SrV_r1: 5′‐CAG CCT GTT ATC CCT AGA G‐3′ (410 pb; Sherwood & Presting, 2007), targeting the 23S rRNA gene. This primer has been successfully used in previous studies (e.g. Hamard *et al*., 2021b; Le Geay *et al*., 2024b). The primers were tagged with Illumina adapters and used for DNA amplification. PCR was conducted in a total volume of 50 μl, consisting of 25 μl of AmpliTaq Gold™ Master Mix (Applied Biosystem, ThermoFisher, Waltham, MA, USA), 19 μl of nuclease‐free ultrapure water, 1 μl of forward and 1 μl of reverse primer (20 μM in final concentration), and 4 μl of DNA. The following PCR conditions were used: 10 min at 95°C (activation), followed by 35 cycles of 60 s at 94°C (denaturation), 45 s at 55°C (annealing), and 45 s at 72°C (elongation), terminated by a final elongation of 10 min at 72°C. The quality of PCR amplicons was assessed on a 1.65% agarose gel electrophoresis, and sequencing was performed using Illumina MiSeq technology (V3 chemistry, 2 × 250 pb) by the GeT‐PlaGe platform (Genotoul, Toulouse, France).

Paired‐end fastq sequences were analysed using the FROGS pipeline (Find Rapidly Operational Taxonomic Units Galaxy Solution) on the Galaxy platform (Escudié *et al*., 2018). Paired‐end reads were merged using Vsearch (10% of mismatch; Rognes *et al*., 2016). Sequences were filtered based on their length (minimum amplicon size: 300 bp, maximum amplicon size: 500 bp), and primer mismatches were removed. Sequences were then dereplicated and clustered into OTUs using the Swarm clustering method with an aggregation distance of three (Mahé *et al*., 2014). Chimeras were identified and removed using Vsearch. Filters were then applied to remove singletons. OTUs were assigned at different taxonomic levels using the *μ*green reference database for the 23S rRNA gene specifically developed for photoautotrophic communities (Djemiel *et al*., 2020). Further analyses were conducted with the phyloseq R package (McMurdie & Holmes, 2013). In particular, we removed plant‐associated sequences (mostly *Sphagnum*) and kept only microbial photoautotrophic OTUs. This was performed by removing all OTUs belonging to the class Embryophyceae. We removed 888 283 sequences, corresponding to 72% of all sequences. To alleviate sequencing artefacts, we then performed a rarefaction for each sample that resulted in 121 794 microbial photoautotrophic sequences (2298 per sample) belonging to 4978 OTUs, on average 229 (135–316) OTUs per sample. Finally, we checked the profiles of the rarefaction curves. At the sample level, most samples either reached saturation or were nearly saturated (Fig. S1). This indicated that the majority of the diversity of the six study areas was captured. However, as all rarefaction curves did not reach saturation, we acknowledge that rare taxa might have been overlooked while our analysis emphasises the most abundant taxa.

#### Photosynthetic community absolute quantification

Absolute quantification of the 23S rRNA gene targeting both eukaryotic and prokaryotic oxygenic photoautotrophs was performed using digital PCR (dPCR; Qiagen) using the primer pair 23S255f/ P23SrV_r1 (Sherwood & Presting, 2007; Le Geay *et al*., 2024b). Reactions were run in a total volume of 12 μl with 4 μl of EvaGreen master mix (Qiagen, 1X), 0.6 μl of each primer (final concentration 0.5 μM), and 2.8 μl of ultrapure water. 4 μl of template DNA diluted 1/10 was added to the reaction mix. This reaction mix was further transferred to a dPCR nanoplate 8.5 k, and dPCR was run in a QIAcuity Digital PCR System with the following cycling conditions: 2 min at 95°C, 20 cycles of 15 s at 95°C, 15 s at 60°C, and 15 s at 72°C, followed by 25 cycles of 15 s at 95°C, 15 s at 58°C, and 15 s at 72°C, finally followed by 5 min at 40°C. Imaging was made for 350 ms with a gain of 3. *Micromonas pusilla* DNA diluted 1/100 was used as a positive control, and ultra‐pure water as a nontarget control (NTC) to set up the threshold separating positive and negative partitions. The final concentration was obtained using the volume of elution (70 μl), the volume of dPCR (12 μl), the volume of template DNA (4 μl), the dilution factor of the template DNA (10), and the amount of dry moss sample used for DNA extraction to obtain the final concentration in target gene copies per gram of dry peat (copies.g^−1^ DW). The absolute abundance of different photoautotrophic groups was then calculated using their relative abundance from 23S rRNA sequencing data.

#### Photosynthetic capacity of the photoautotrophic microbial community

To estimate the microbial photosynthetic capacity, a sample of the topmost 3 cm of dominant mosses was collected next to each permanent sample plot in summer 2022. The dominant moss species were *Sphagnum* spp. in all but two sample plots in the rich fen WLD area that were dominated by *Dicranum polysetum*. The samples were packed in ziplock bags with some air and stored cold (for 3 d before analyses).

In the laboratory, we first extracted the microbes from mosses by placing the moss sample in 30 ml demineralised water and shaking it gently by hand for 1 min and 30 s on a vortex. Then, we squeezed the moss in a strainer to obtain our microbial solution. The remaining moss remnants were removed by filtering the samples through 100 μm sieves, and the microbial community was retained by filtering the solution through GF/F Whatman filters (0.7 μm). The filters containing the microbes were then kept dark for 30 min, after which we used a PhytoPAM (Phytoplankton Analyzer, Heinz Walz GMBH, Effeltrich, Germany) to measure the quantum yield of photosystem II (φPSII) and ETR_max_ by the rapid light–response curves method (Hamard *et al*., 2021a). φPSII gives the fraction of the absorbed quanta that are used for photosynthetic electron transport and thus provides a measure of photosynthesis efficiency, while ETR_max_ gives the maximum electron transport rate through photosystem II and thus provides an indication of the maximum photosynthetic activity of the microbial community (Maxwell & Johnson, 2000).

#### Photosynthetic activity of the bryosphere

Bryospheric photosynthesis was calculated as community‐weighted mean (CWM) photosynthetic capacity, and includes photosynthesis conducted by both mosses and associated microbes existing in each sample plot. Bryospheric photosynthesis of the most common moss species at each site was measured from a sample of several capitula set on a cuvet of an infrared gas analyser (LI‐6400; Li‐Cor Inc., Lincoln, NE, USA and GFS‐3000, Walz, Germany) exposed to four light levels: 1500, 250, 35, and 0 μmol m^−2^ s^−1^. Photosynthetic capacity (*P*_max_) was based on light–response curve modelling. The methodology is explained in detail in Kokkonen *et al*. (2022).

To estimate the CWM *P*_max_ for each sample plot from which the microbial samples were taken, moss species cover was estimated in summer 2021 (Köster *et al*., 2023) CWM P_max_ was calculated for each sample plot with the bat package in R environment (Cardoso *et al*., 2022) based on species cover and species‐specific *P*_max_ values.

### Statistical testing

All statistical analyses of the data were conducted in Rstudio (v.2022.07.2) in R environment (v.4.2.2; R Core Team, 2022). For visualisation, we used the package ggplot2 (Wickham, 2016) and cowplot (Wilke, 2024).

To test whether the site‐specific effect of WLD on the photosynthetic parameters (ETR_max_, φPSII, *P*_max_), environmental variables, decomposition (*k*_TBI_), alpha diversity, and the absolute abundance of the dominant photoautotrophic phyla, we ran a two‐way ANOVA followed by Tukey's *post hoc* test. Shannon–Wiener and Simpson indices were converted to show the effective number of OTUs, that is exp(*H*) and 1/*D*, and evenness was counted using the effective Shannon–Wiener index (Jost, 2006). When necessary, these data were cubic root or log‐transformed to meet the parametric assumptions. If the assumption of the homogeneity of variances was still not met, the nonparametric Scheirer–Ray–Hare test was used. If these data were not normally distributed, but variances were equal, we made sure that the residuals of the linear model were normally distributed and had equal variances before using ANOVA. Results from all two‐way ANOVAs and the respective Scheirer–Ray–Hare test are compiled in Table S2.

The variation in the community composition of photoautotrophic microbial OTUs was visualised with nonmetric multidimensional scaling (NMDS) in the package vegan (Oksanen *et al*., 2022), using absolute abundances of each OTU as input data. To assess the differences among treatments and sites, we applied permutational multivariate ANOVA (*adonis*). The differences in the multivariate homogeneity of group dispersions were assessed with the function *betadisper*, and the extent to which the microbial phyla and orders (families for cyanobacteria) explained the community composition identified by NMDS was tested with *envfit*.

To assess the impact of the treatment and the site on the community composition of the various taxonomic levels (i.e. Phylum, Order, Genus), multivariate generalised linear models were built using negative binomial distributions with package mvabund (Wang *et al*., 2012). For each taxonomic level, we used the absolute abundances including all taxa occurring in more than two plots. However, in the univariate result tables (Tables S3, S4), statistics are only shown for phyla, orders, and genera with a relative abundance > 0.01%.

We used linear models to explain the variation in the photosynthetic parameters. We inspected photosynthetic parameters in relation to decomposition rate *k*_TBI_, the absolute abundance of the dominant photoautotrophic phyla and all photoautotrophic microbes, and selected environmental variables (Table S1) and their combined metrics; that is, the first principal component (PC1) derived from a principal component analysis performed on the environmental variables with the package vegan (Oksanen *et al*., 2022). The PC1 values represented the combined effect of highly correlated fertility and pH. Logarithmic transformation was used when needed. The relative importance of different explanatory variables was assessed by comparing linear models with different explanatory variables using Akaike's Information Criterion (AICc values from aictab‐function in the package aiccmodavg, (Mazerolle, 2023)). We first built models with a single explanatory variable and added more variables to the best model until the model performance did not improve from additional variables.

**Fig. 2:**
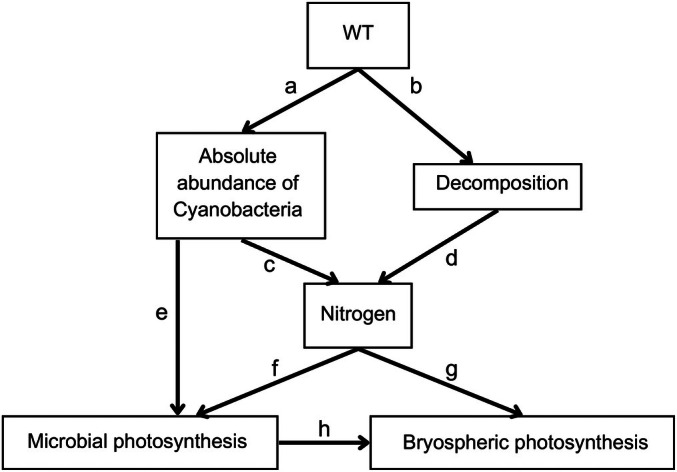
Hypothesised connections between environmental variables, microbial community, and photosynthesis. This model was used as an *a priori* model for both control and water level drawdown datasets in our structural equation model analysis. The letters indicate connections described in Table 1. WT, water table depth.

To understand how the persistent drying changes processes controlling microbial and bryospheric photosynthesis, we conducted structural equation models using the package piecewisesem (Lefcheck, 2016). We used structural equation model (SEM) to specifically test how nitrogen provided by Cyanobacteria and decomposition promotes photosynthesis and how these linkages are influenced by wild‐table in pristine and disturbed (WLD) conditions. Two separate models were built: One included samples from the control areas, and the other included samples from the WLD areas. We used the same *a priori* model for both control and WLD datasets. The *a priori* model is presented in Fig. 2, and the connections tested are listed in Table 1. Within the SEMs, we used linear models. Variables were excluded or included based on the suggestions of the model outputs, and the models were evaluated based on Fisher's *C*‐values and Chi‐squared values (Table S5). Finally, we compared the two SEMs in order to assess how WLD has changed processes that control photosynthesis.

**Table 1:** List of connections tested with the structural equation model (SEM), along with related references.

Arrow in Fig. 2	Explanatory variable	Response variable	Hypothesis	References
a	Water table (WT) depth	Absolute abundance of Cyanobacteria	The abundance of Cyanobacteria increases as WT deepens, as they are adapted to dry conditions.	Nichols & Adams (1982); Osborne & Raven (1986); Perrine *et al*. (2012)
b	Water table depth	Potential decomposition rate	Decomposition potential is higher when WT is deeper.	For example Laiho (2006) and references therein
c	Absolute abundance of Cyanobacteria	Soil nitrogen concentration	A high abundance of Cyanobacteria increases soil nitrogen concentration	For example Larmola *et al*. (2014); Carrell *et al*. (2022b)
d	Decomposition	Soil nitrogen concentration	A higher potential decomposition rate increases soil nutrient concentrations	Holden *et al*. (2004) and references therein; Munir *et al*. (2017)
e	Absolute abundance of Cyanobacteria	ETR_max_	Cyanobacteria photosynthesise and, therefore, their abundance promotes microbial photosynthesis.	Hamard *et al*. (2021b)
f	Soil nitrogen concentration	ETR_max_	Soil nitrogen concentration promotes microbial photosynthesis.	Wyatt & Turetsky (2015); Gu & Wyatt (2016); DeColibus *et al*. (2017)
g	Soil nitrogen concentration	*P*_max_	Soil nitrogen concentration promotes bryospheric photosynthesis	Limpens *et al*. (2011); Granath *et al*. (2012); Berg *et al*. (2013)
h	ETR_max_	*P*_max_	Increased microbial photosynthesis contributes to an increase in bryospheric photosynthesis.	Hamard *et al*. (2021a); Jassey *et al*. (2022a,b)

Each row corresponds to an arrow in our *a priori* SEM (Fig. 2).

## Results

### Environmental variables and decomposition potential

**Fig. 3:**
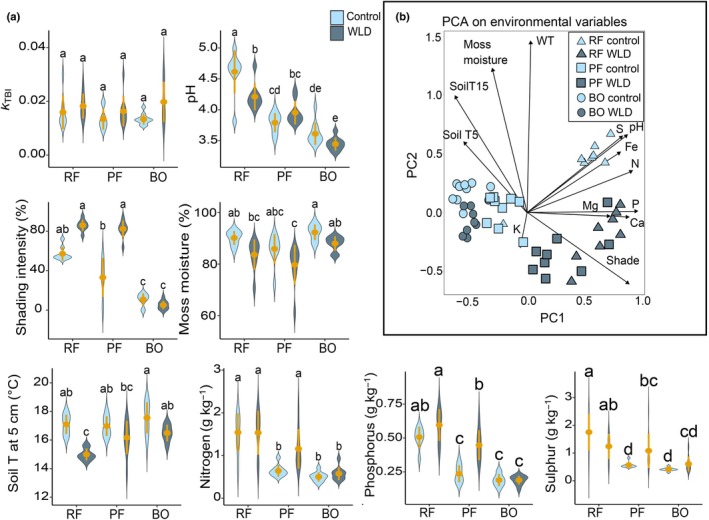
Abiotic characteristics of the study areas in the Lakkasuo water level drawdown experiment. RF, rich fen; PF, poor fen; BO, bog; WLD, water level drawdown. (a) Decomposition rate *k*_TBI_ and environmental variables. The violins illustrate the distribution of the data and the kernel probability density of the data at different values. The yellow dots indicate mean values, and the whiskers show SD. Letters indicate statistically significant differences Tukey's pairwise comparison (*P*‐value <0.05). Soil T at 5 cm = Soil temperature at 5 cm depth. (b) A principal component analysis of the environmental variables in the study set‐up. 48% of the variation in the environmental variables was related to the study site, 26% to the WLD treatment, and 79% to their interaction. WT, water table depth, Soil T5/T15 = Soil temperature at 5/15 cm depth. P, Mg, S, Fe, Ca, and N refer to the concentration of the corresponding element.

The control and WLD areas, and the three sites (rich fen, poor fen, bog) differed from each other regarding the measured environmental variables (Fig. 3a; Table S2). WLD areas were typically more shaded, cooler, drier, and richer in nutrients than the control areas. This was seen in the PCA of environmental variables, where all sites differed from each other and were ordered along the PC1 from bog to rich fen following the increasing nutrient gradient (Fig. 3b). The WLD‐induced increase in shading and soil nutrient concentrations had shifted the poor fen towards the higher end of the nutrient gradient (Fig. 3b). The decomposition rate *k*_TBI_ varied between 0.009 and 0.03. It was significantly higher in WLD areas than in control areas, but not affected by site, nor was there an interaction between the effects of site and treatment (Fig. 3a; see Table S2 for statistical justification).

### The abundance and taxonomic composition of photoautotrophic microbes

**Fig. 4:**
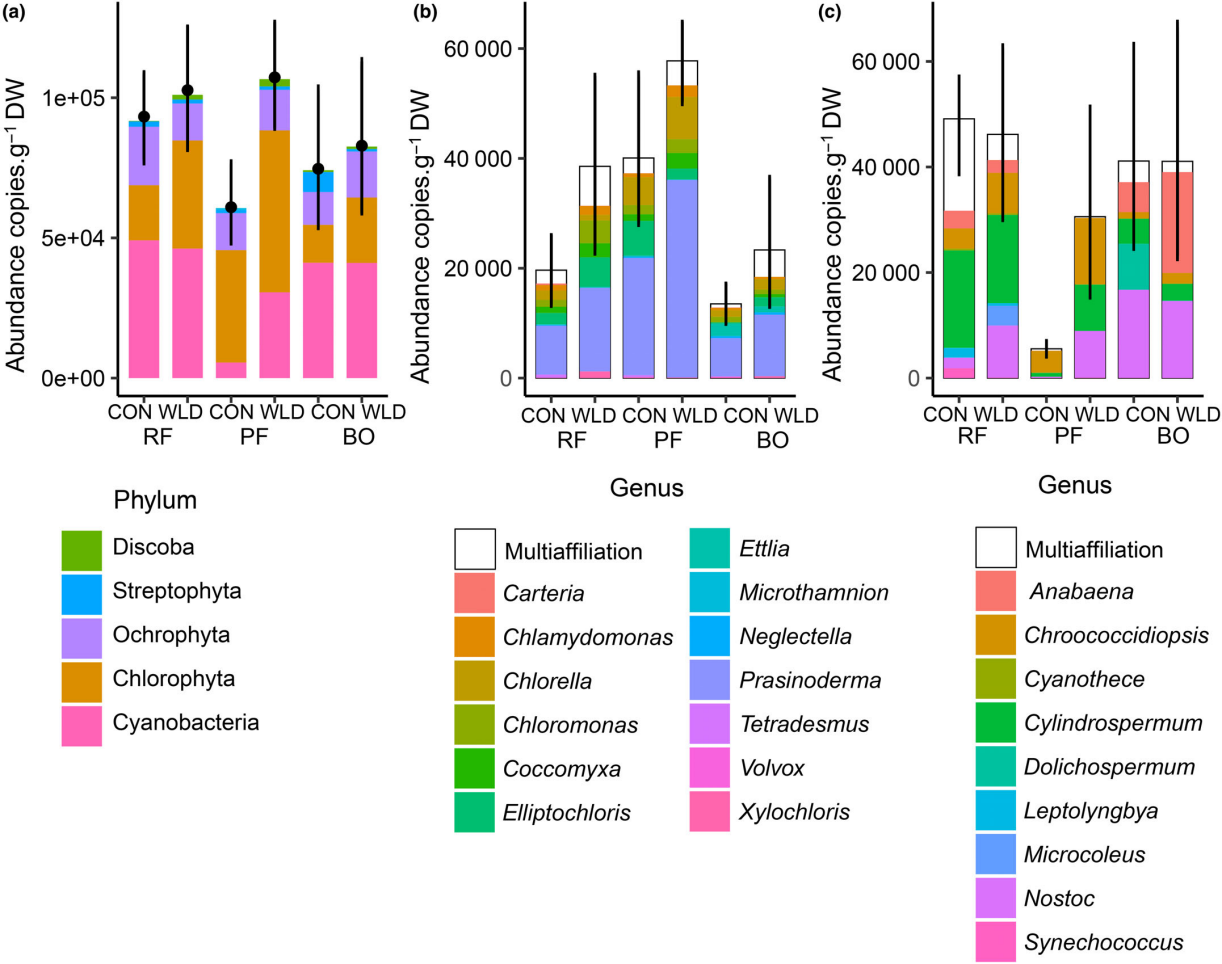
Absolute abundances (as gene copies per 1 g of dry moss) of photoautotrophic microbes in the study areas of Lakkasuo experiment (CON, control area; WLD, water level drawdown area; RF, rich fen; PF, poor fen; BO, bog). (a) Five most abundant photoautotrophic phyla. The excluded phyla were Cercozoa, Cryptophyta, Dinoflagellata, Glaucophyta, Haptophyta, and Rhodophyta, whose maximal coverages were 0.03%, 0.5%, 0.08%, 0.4%, and 1.2%, respectively. The black dot indicates the average total abundance of photoautotrophic microbes, and the lines show the variation in each study area. (b) Absolute abundance of the most abundant genera in the phylum Chlorophyta. The lines show the variation of total photoautotrophic abundance in each study area. (c) Absolute abundance of the most abundant genera in the phylum Cyanobacteria. The lines show the variation of total photoautotrophic abundance in each study area.

The sequenced photoautotrophic microbial community consisted of 10 eukaryotic phyla and one prokaryotic phylum, namely Cyanobacteria (Fig. 4). The most abundant phyla were Chlorophyta (22–56%), Cyanobacteria (10–54%), and Ochrophyta (14–23%), together constituting, on average, over 90% of the community in all study areas. Over 92% of the Cyanobacteria belonged to the order Nostocales. Chlorophyta and Ochrophyta consisted of 13 and 10 orders, respectively. The most common Chlorophyta order was Prasinococcales, followed by Chlamydomonadales, Chlorellales, and Prasiolales. Ochrophyta assemblages were dominated by Bacillariophyta (diatoms), Eustigmatophyceae, and Synurales.

**Fig. 5:**
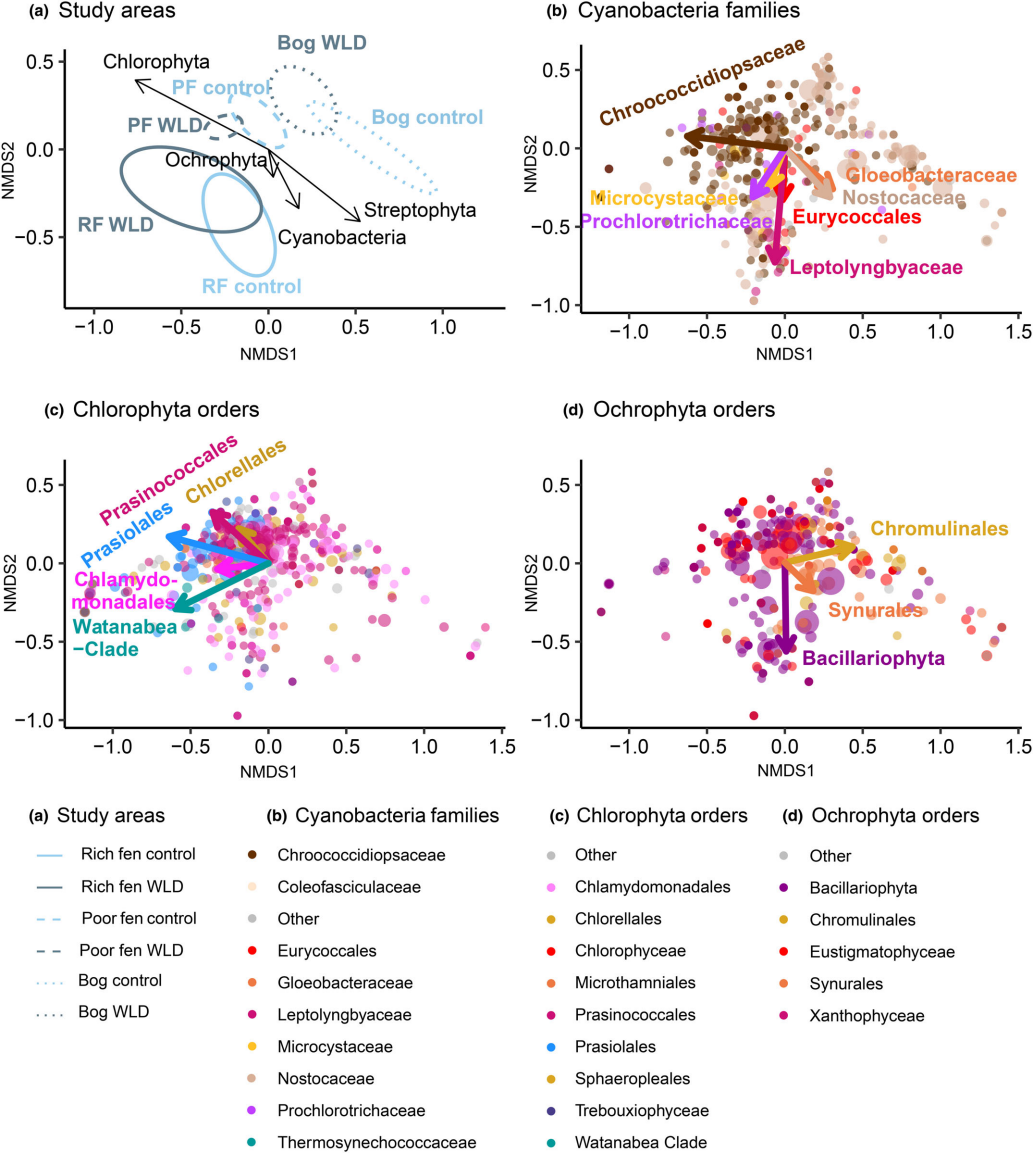
Nonmetric multidimensional scaling (NMDS) ordination based on the absolute abundance of all photoautotrophic OTUs in the sampling points. Frame (a) shows the study areas as ellipses based on all photoautotrophic OTUs, and the vectors show the directions towards which the abundances of the most abundant phyla increase (see *r* ^2^ values and significances in Supporting Information Table S7). RF, rich fen; PF, poor fen; WLD, water level drawdown. Frames (b)–(d) are based on the same ordination analysis as Frame (a), but they visualise different photoautotrophic groups: Cyanobacteria families (b), Chlorophyta orders (c), and Ochrophyta orders (d). The size of a dot reflects the absolute abundance of the OTU. The vectors represent those orders and families that explained the distribution of photoautotrophs along the two NMDS axes (see the full list of *envfit* results in Table S7). Note that orders and families that did not respond to the treatment, or whose relative abundance was < 0.01%, are included as ‘Others’.

WLD affected the overall taxonomic composition of the photoautotrophic community, and this became more evident in the lower taxonomic levels (Table S6; Fig. 4). The result was confirmed by two‐way permutational multivariate ANOVA (*adonis*; *P*‐value = 0.001; Fig. 5). Site:Treatment interaction was significant (*P*‐value = 0.002); however, *pairwise adonis* showed relatively similar treatment effects in the three sites: *F* (and *P*‐values) of 2.04 (0.019), 2.9 (0.002), and 2.5 (0.008) for the rich fen, the poor fen, and the bog, respectively. The total absolute abundance of photoautotrophic microbes increased with WLD, especially in the poor fen (Fig. 4a; Table S2). The absolute abundance of Chlorophyta, Cyanobacteria, Dinoflagellata, and Discoba increased in WLD areas compared with control areas, particularly for Cyanobacteria in the poor fen. Three Chlorophyta orders (Microthamniales, Prasinococcales, and Watanabea Clade) and two Cyanobacteria orders (Nostocales and Synechococcales) benefited from WLD, while one Chlorophyta order (Sphaeropleales), one Cyanobacteria order (Leptolyngbyales), and four Ochrophyta orders (Chromulinales, Eustigmatophyceae, Sarcinochrysidales, and Synurales) preferred control areas. The absolute abundance of 38 genera also had a significant response to WLD, at least in some sites (Tables S3, S4, S6). From the most abundant Cyanobacteria genera, WLD was preferred by *Chroococcidiopsis*, *Nostoc* in rich fen, and *Prochlorotrix* in both fens, and from the most abundant Chlorophyta genera, by *Prasinoderma*, *Elliptochloris* in rich fen, *Coccomyxa*, and *Chlamynomodas*. The alpha diversity (OTU richness, community diversity, and evenness) of photoautotrophic communities was unaffected by WLD (Fig. S2; Table S2). Similarly, the variation within study areas (from *betadisper*) was unaffected by WLD (*P*‐value = 0.98).

Two‐way permutational multivariate ANOVA (*adonis*) and MANOVA (Fig. 5; Tables S6, S7) showed that photoautotrophic communities differed from each other between the sites (*P*‐values = 0.001). The poor fen was dominated by eukaryotes, and both the rich fen and the bog had an almost even ratio of eukaryotes and prokaryotes (Cyanobacteria). Chlorophyta were typical of the poor fen (driven by the orders Chlorellales, Prasiolales, and Prasinococcales), while Cyanobacteria were characteristic in the bog and the rich fen: Nostocales in both, Leptolyngbyaceae and Eurycoccales in the rich fen, and Gloeobacteria in the bog. Ochrophyta was common across all sites, but the order Bacillariophyta (diatoms) preferred the rich fen (Figs 4, 5). Also, both alpha and beta diversity varied between the sites. OTU richness decreased from the rich fen to the bog, whereas both the diversity and evenness peaked in the poor fen but did not differ between the rich fen and the bog (Fig. S2; see Table S2, for statistical justification). The beta diversity quantified with the function *betadisper*, that is OTU variation within study areas, was significantly lower in the poor fen than in the other two sites (*P*‐value = 0.006; Fig. 5).

### Microbial and bryospheric photosynthetic capacity

**Fig. 6:**
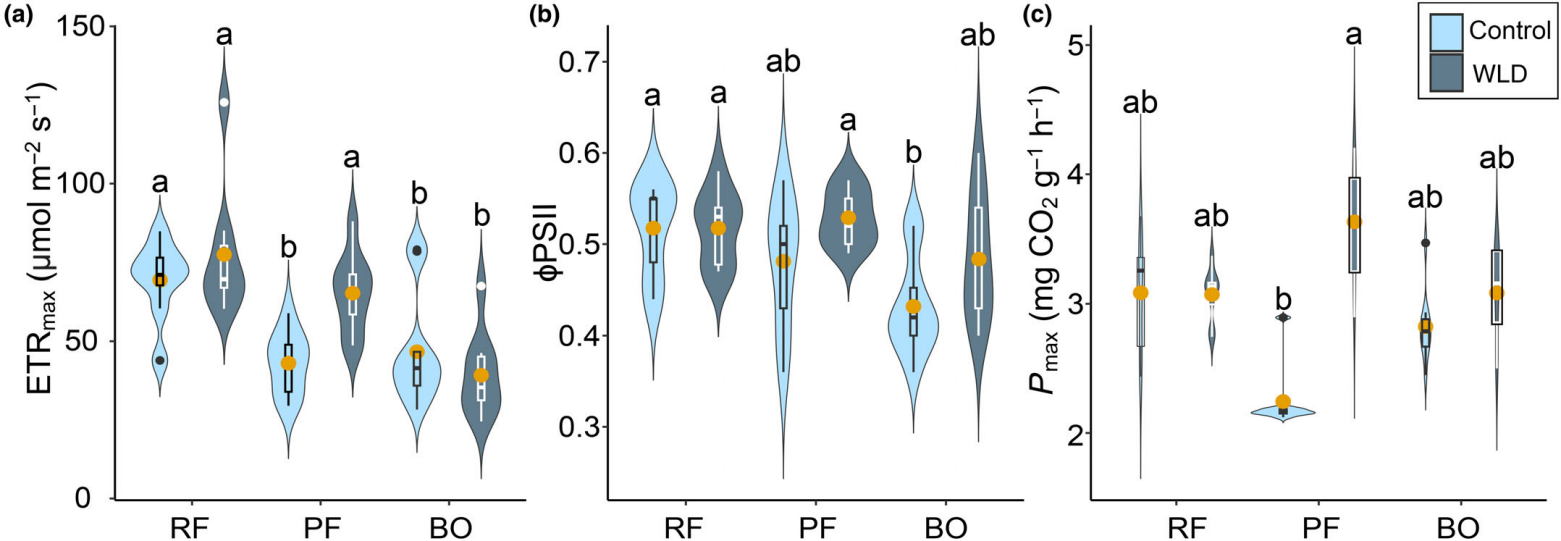
Photosynthetic parameters in the study areas of Lakkasuo water level drawdown experiment (RF, rich fen; PF, poor fen; BO, bog; WLD, water level drawdown). The violins illustrate the distribution of the data and the kernel probability density of the data at different values. The median and interquartile range are shown within the violin distribution as box plots. The yellow dots show the mean value. Letters indicate significant differences according to Tukey's pairwise comparison (*P*‐value < 0.05). (a) Microbial photosynthetic capacity measured as maximum electron transport rate (ETR_max_). (b) Microbial photosynthetic efficiency measured as quantum yield of photosystem II (φPSII). (c) Bryospheric photosynthetic capacity measured as *P*_max_.

The proxy for the photosynthetic activity, measured as ETR_max_, varied from 24 to 126 μmol m^−2^ s^−1^. The main effect of WLD was insignificant, but ETR_max_ was significantly higher in the poor fen WLD area than in the control area. It also increased along with the fertility gradient, being significantly higher in the rich fen than in the two other sites (Fig. 6a; see Table S2, for statistical justification). ETR_max_ was best explained by soil phosphorus concentration, quantum yield of photosystem II, and the absolute abundance of Chlorophyta. Together, they explained 61% of the variation. When models with a single explanatory variable were compared, phosphorus was the best environmental variable explaining ETR_max_ (Table 2; Fig. S3).

**Table 2:** Comparison of linear models with different explanatory variables for microbial photosynthesis (ETR_max_, φPSII) and bryospheric photosynthesis (*P*_max_).

Model	*K*	AICc	Δ__AICc	AICcWt	Cum.Wt	LL	Adj. *R* ^2^	*F*_df_	*P*
**ETR**_**max**_ **~ P + φPSII + Chlorophyta**	**5**	**2.9**	**0**	**0.12**	**0.12**	**4.23**	**0.609**	**26.4**	**< 0.001**
ETR_max_ ~ P + φPSII + Chlorophyta + Mg	6	2.92	0.02	0.12	0.23	5.52			
ETR_max_ ~ P + φPSII + Cyanobacteria	5	3.29	0.4	0.1	0.33	4.04			
ETR_max_ ~ P + φPSII + Chlorophyta + S	6	3.6	0.7	0.08	0.41	5.18			
ETR_max_ ~ P + φPSII + Chlorophyta + Fe	6	4.41	1.51	0.06	0.47	4.77			
ETR_max_ ~ P + φPSII + Chlorophyta + Shade	6	4.53	1.64	0.05	0.52	4.71			
ETR_max_ ~ P + φPSII + Chlorophyta + *k*_TBI_	6	4.53	1.64	0.05	0.57	4.73			
ETR_max_ ~ P + φPSII + Chlorophyta + pH	6	4.63	1.73	0.05	0.62	4.66			
ETR_max_ ~ P + φPSII + Chlorophyta + N	6	4.65	1.76	0.05	0.67	4.65			
ETR_max_ ~ P + φPSII + Chlorophyta + WT	6	4.67	1.77	0.05	0.72	4.64			
**φPSII ~ PC1 + WT**	**4**	**−156.7**	**0**	**0.32**	**0.32**	**82.77**	**0.277**	**10.97**	**< 0.001**
φPSII ~ PC1 + WT + Shade	5	−156.67	0.03	0.32	0.64	83.97			
φPSII ~ PC1 + WT + Moss moisture	5	−154.87	1.83	0.13	0.76	83.07			
***P***_**max**_ **~ Cyanobacteria + WT + S + *k***_**TBI**_	**6**	**61.1**	**0**	**0.12**	**0.12**	**−23.55**	**0.425**	**9.87**	**< 0.001**
*P*_max_ ~ Cyanobacteria + WT + S	5	61.83	0.73	0.09	0.21	−25.24			
*P*_max_ ~ Cyanobacteria + WT + pH	5	62.18	1.08	0.07	0.28	−25.41			
*P*_max_ ~ Cyanobacteria + WT + S + *k*_TBI_ + Fe	7	62.3	1.2	0.07	0.35	−22.79			
*P*_max_ ~ Cyanobacteria + WT + S + *k*_TBI_ + pH	7	62.4	1.3	0.06	0.41	−22.83			
*P*_max_ ~ Cyanobacteria + WT + S + *k*_TBI_ + P	7	62.55	1.45	0.06	0.47	−22.91			
*P*_max_ ~ Cyanobacteria + WT + S + Fe	6	62.78	1.67	0.05	0.53	−24.41			
*P*_max_ ~ Cyanobacteria + WT + N	5	62.87	1.77	0.05	0.58	−25.75			
*P*_max_ ~ Cyanobacteria + WT + S + P	6	63.02	1.92	0.05	0.63	−24.54			

The best models are in bold, based on AICc ranking. The table also presents the number of estimated parameters (*K*), the difference in AICc relative to the best model (ΔAICc), the probability of the given model being the best among all tested models (AICcWt), cumulative Akaike weights (Cum. Wt), and the model's maximum likelihood estimation (LL). For the best models, the adjusted *R* ^2^ value and *F*‐statistics are also shown. The table lists only the models with ΔAICc < 2. Explanatory variables are as follows: Chlorophyta, absolute abundance of Chlorophyta; Cyanobacteria, absolute abundance of Cyanobacteria; Shade, shading intensity; *k*_TBI_, decomposition rate *k* quantified by Tea Bag Index; WT, water table depth; moss moisture, moss moisture content (% of weight). P, Mg, S, Fe, and N refer to the concentration of the corresponding element. See the correlations between individual variables in Supporting Information Fig. S3.

The proxy for the condition of the photosynthetic machinery, φPSII, ranged from 0.36 to 0.6. It increased significantly both following WLD and along with the fertility gradient, being higher in the two fens than in the bog (Fig. 6b). As there was no interaction between the site and the treatment, the effect of WLD on φPSII was not site‐dependent. The best driver of φPSII was the combination of water table depth and PC1 axis of environmental conditions that represented the combined effect of fertility and pH. Together, the water table depth and PC1 explained 28% of the observed variation (Table 2; Fig. S3).

Bryospheric photosynthetic capacity, *P*_max_, was elevated following WLD; however, Tukey's *post hoc* test revealed that the difference between the WLD and control area was significant only in the poor fen (Fig. 6c). *P*_max_ was best explained by the absolute abundance of Cyanobacteria, WT, soil sulphur concentration, and decomposition rate *k*_TBI_. These variables explained 43% of the variation in *P*_max_ (Table 2; Fig. S3).

### Interplay between photosynthesis, decomposition, and environmental variables

**Fig. 7:**
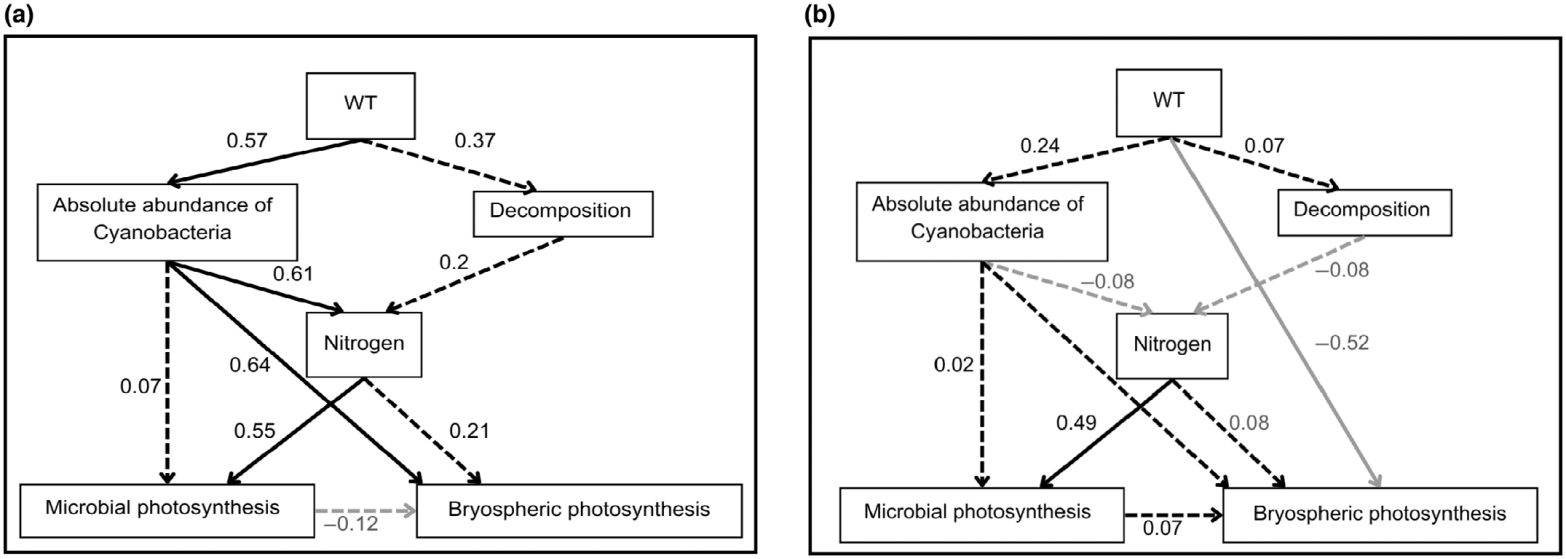
Structural equation models showing connections between factors and processes in control areas (a) and water level drawdown areas (b). Black arrows indicate a positive impact, and grey arrows a negative impact; solid arrows a significant impact, and dashed arrows non‐significant impacts in the final model. Standardised estimates are given next to each arrow. See model diagnostics in Supporting Information Table S5. WT, water table depth.

A SEM built on the samples from control areas (Fig. 7a) showed that in control areas, the water table closer to the peat surface promoted the abundance of Cyanobacteria, and that the high abundance of Cyanobacteria explained the high soil nitrogen concentration, which promoted microbial photosynthesis, here quantified as ETR_max_. While the soil nitrogen concentration did not significantly explain bryospheric photosynthesis, the abundance of Cyanobacteria did. On the contrary, in WLD areas (Fig. 7b), the data did not reflect such connections, but deep WT directly promoted bryospheric photosynthesis, while the soil nitrogen concentration promoted microbial photosynthesis.

## Discussion

In this study, we investigated how two decades of moderate WLD have changed photoautotrophic microbial communities and the photosynthetic capacity of both microbes and the entire moss layer, bryosphere. While we found significant changes in the photoautotrophic community in all sites, the photosynthetic capacity of both microbes and the bryosphere was increased in the poor fen only. We found that the most important drivers of photosynthetic activity differed between microbial photosynthesis and bryospheric photosynthesis and were altered by long‐term drying.

### Photoautotrophic microbial communities as impacted by water level drawdown

In agreement with our hypothesis (H1), the photoautotrophic communities associated with mosses in the three sites responded to the WLD at phylum, order, and genus levels. Contrary to Hypothesis 3, the responses did not show the expected pattern of the largest changes in the rich fen and the smallest changes in the bog.

The total absolute abundance of photoautotrophic microbes increased after WLD. As we expected (H1), Cyanobacteria abundance increased after WLD, especially in the poor fen, but so did the other large phylum, Chlorophyta. This was driven by the highly abundant orders Nostocales and Prasinococcales, and within them, genera *Chroococcidiopsis* and *Prasinoderma*, both of which benefited from WLD. However, both Cyanobacteria and Chlorophyta had heterogenic responses to WLD at the genus level; some genera gained and some lost abundance after WLD. This likely explains the controversy between SEM showing that Cyanobacteria were generally promoted by WT close to the peat surface, and the observed abundance increase following WLD in the driest site, poor fen. A major proportion of Cyanobacteria in the poor fen belonged to the genus *Chroococcidiopsis*, which is known to tolerate extreme conditions (e.g. Billi *et al*., 2000; Baldanta *et al*., 2023). Nevertheless, Chlorophyta, Cyanobacteria, and Ochrophyta remained dominant in all study areas. While these phyla have been found in high abundances in peatlands throughout Europe (Hamard *et al*., 2021a) and in Alaska (DeColibus *et al*., 2017), the result is in slight contrast with previous studies that report higher sensitivity of the dominant phyla to hydrological conditions (Rober *et al*., 2013; Hamard *et al*., 2021b). Even though the community composition was affected by WLD, the diversity of these communities was unaffected by WLD.

### Microbial and bryospheric photosynthesis as impacted by water level drawdown

Both microbial (ETR_max_) and bryospheric (*P*_max_) CO_2_ fixation rates increased in the poor fen WLD area but were unaffected in the rich fen and the bog, partly supporting Hypothesis 2. However, contrary to Hypothesis 3, the strength of the response did not follow a similar pattern as previously found for the vegetation (increase from bog to rich fen; Kokkonen *et al*., 2019b; Laine *et al*., 2021). While increased microbial carbon fixation in relatively dry conditions has been suggested (DeColibus *et al*., 2017; Hamard *et al*., 2021b; Le Geay *et al*., 2024a), stable microbial CO_2_ fixation rates have also been reported across varying hydrological conditions and communities (Hamard *et al*., 2021a; Jassey *et al*., 2022a). Both increased and stable bryospheric photosynthesis in drying conditions have been reported before (Hájek *et al*., 2009; Kangas *et al*., 2014; Kokkonen *et al*., 2022). In all study sites, WLD increased φPSII, indicating that the microbes were exposed to lower stress and used light more efficiently in more shaded and drier conditions. As φPSII reflects the condition of photosystem II, this agrees with previous findings suggesting the properties of the light‐harvesting mechanisms as an adaptation mechanism (Perrine *et al*., 2012). This result was similar to that previously found for *Sphagnum* mosses (Hájek *et al*., 2009).

We note that our estimates of microbial photosynthesis are based on several assumptions that include uncertainty. For example, while our extraction method has been previously used successfully (Hamard *et al*., 2021a,b), it may not catch all endophytic Cyanobacteria, whose abundance could thus be slightly underestimated. We also acknowledge that our fluorescence‐based measurements of ETR_max_ could introduce some bias in our results. Chl fluorescence measurements often underestimate the photosynthetic potential of prokaryotes, such as Cyanobacteria (Schuurmans *et al*., 2015; Ogawa *et al*., 2017), leading to a potential underestimation of photosynthetic rates in the sites with a high proportion of Cyanobacteria.

### Drivers of microbial and bryospheric photosynthesis

Bryospheric and microbial CO_2_ fixation rates were driven by partly different variables: bryospheric photosynthesis (*P*_max_) was driven by deep WT, high soil sulphur concentration, high decomposition potential, and high abundance of Cyanobacteria, while microbial CO_2_ fixation (ETR_max_) was driven by high soil phosphorus concentration, the condition of photosystem II within photoautotrophic microbes (φPSII), and high Chlorophyta abundance. As soil nutrient concentrations and/or pH were important drivers of microbial photosynthesis (ETR_max_ and φPSII), both microbial photosynthetic parameters increased along the site fertility gradient (from bog to rich fen), but this was not seen for bryospheric photosynthesis. While earlier findings suggested that both nitrogen and phosphorus limit microbial production (Wyatt & Turetsky, 2015; Gu & Wyatt, 2016; DeColibus *et al*., 2017), our data highlighted the importance of phosphorus. Microbial CO_2_ fixation benefited indirectly from deep WT, since it promoted φPSII that further explained high ETR_max_. The higher φPSII, that is better condition of photosystem II within the microbes in the WLD areas, suggests that moderate drying promotes microbial photosynthesis through intracellular adaptation.

Decomposition was accelerated in all WLD areas, likely explaining the increased soil nutrient concentrations in WLD areas (Laiho, 2006). Thus, the distinct increase in microbial and bryospheric CO_2_ fixation rate in the poor fen WLD area may be explained by the deeper WT and the consequently larger increase in soil phosphorus and sulphur concentration compared with the other two WLD areas. While sulphur was the only nutrient whose concentration directly promoted bryospheric photosynthesis, the beneficial effects of decomposition rate and Cyanobacteria were also likely related to nutrient mineralisation and nitrogen fixation, respectively. Earlier findings have revealed that a small increase in nitrogen concentration tends to be beneficial in nitrogen‐limited systems, such as boreal peatlands (Limpens *et al*., 2011; Granath *et al*., 2012; Berg *et al*., 2013). The indicated importance of sulphur was possibly related to its role as a key element in proteins needed in photosynthesis and a facilitator in the uptake of other nutrients (Warshan *et al*., 2017; Carrell *et al*., 2022b); however, our study calls for further research in the role of sulphur in bryospheric photosynthesis.

### Interplay between photosynthesis, decomposition, and environmental variables as impacted by water level drawdown

In the control areas, we found a connection between Cyanobacteria abundance and soil nitrogen concentration, which further promoted microbial photosynthesis. The tested linkages between soil nitrogen concentration and bryospheric photosynthesis, or microbial and bryospheric photosynthesis, did not get statistically confirmed, but Cyanobacteria abundance directly promoted bryospheric photosynthesis, confirming the results of linear model comparisons. Even though not statistically proven, the positive effect of Cyanobacteria on bryospheric photosynthesis might be based on nitrogen provision to the moss (e.g. Larmola *et al*., 2014; Carrell *et al*., 2022b) and/or promoting higher microbial photosynthesis (Hamard *et al*., 2021b). The result could also indicate that there might be complex and, for now, unknown interactions between Cyanobacteria and the host plant. Overall, the results indicated that Cyanobacteria support bryospheric photosynthesis through nitrogen acquisition, at least in pristine boreal peatlands. On the contrary, such linkages were not found in the WLD treatments. Instead, the result indicated that bryospheric photosynthesis was directly promoted by deep WT in WLD areas, agreeing with previous results that have shown increased bryospheric photosynthesis in dry habitats (Hájek *et al*., 2009; Kangas *et al*., 2014; Kokkonen *et al*., 2022). The role of nitrogen in regulating ETR_max_ was found also in WLD areas, agreeing with previous studies (Wyatt & Turetsky, 2015; Gu & Wyatt, 2016; DeColibus *et al*., 2017). This indicates that WLD could promote higher microbial photosynthesis if soil nutrient concentrations increase either due to accelerated mineralisation and/or increased nitrogen fixation by Cyanobacteria. In future research, studying the active fraction of the photoautotrophic microbial community, that is through rRNA or transcript‐based methods, could enable the discovery of more associations between microbial photosynthesis and the community than current DNA‐based abundance analyses, as the measured φPSII and ETR_max_ reflect only photosynthetically active microbes, but DNA‐based abundance may include inactive cells.

Overall, our study showed Chlorophyta, Cyanobacteria, and Ochrophyta dominated photoautotrophic microbial communities both in drying and pristine boreal peatlands and across the fertility range. Nevertheless, when inspected on deeper taxonomic levels, photoautotrophic microbes had versatile responses to peatland drying, depending on the peatland type and the photoautotrophic group. Furthermore, our study indicated that both microbial and bryospheric photosynthetic rates increased or remained stable after decadal‐scale drying and associated habitat changes. While Cyanobacteria were found to be important in promoting bryospheric photosynthesis in pristine boreal peatlands, the increase in bryospheric photosynthesis was better explained by the direct, beneficial effect of a moderately deep water table. Furthermore, Chlorophyta appeared to be a key taxon driving microbial photosynthesis in all sites and conditions. Overall, the results suggested that photoautotrophic microbes can adapt to drying peatlands through better conditions of photosystem II, community turnover, and an increase in total abundance.

## Competing interests

None declared.

## Author contributions

E‐ST and AML conceived the experiment. OK‐R collected the data under the supervision of E‐ST, VEJJ and JMB. MLG conducted microbial DNA extraction and molecular biology work. OK‐R, HY and VEJJ performed the data analysis. The first manuscript draft was written by OK‐R. All coauthors read and commented on the final version.

## Disclaimer

The New Phytologist Foundation remains neutral with regard to jurisdictional claims in maps and in any institutional affiliations.

## Supporting information


**Fig. S1** Rarefaction curves in each study area.
**Fig. S2** Diversity indices from photoautotrophic microbial communities.
**Fig. S3** Correlations between the photosynthetic parameters and variables that best explained them according to the comparison of linear models (see Table 2, for statistical details.).
**Table S1** Summary of the measured environmental variables.
**Table S2** Statistical comparison of environmental variables, decomposition parameters, photosynthetic parameters, diversity indices, and the absolute abundance of the dominant photoautotrophic phyla between the water level treatments and the three different study sites.
**Table S3** Univariate test results on the impact of water level drawdown, site, and their interaction on the most abundant (relative abundance ≥ 0.01%) photoautotrophic phyla and orders.
**Table S4** Univariate test results on the impact of water level drawdown, site, and their interaction on the most abundant (relative abundance ≥ 0.01%) photoautotrophic genera.
**Table S5** Model diagnostic statistics for structural equation models.
**Table S6** Results of the multivariate generalised linear model on the impact of water level drawdown, site, and their interaction on the different taxonomic levels of the photoautotrophic community.
**Table S7** Explaining power of different taxonomic groups in the non‐metric multidimensional scaling based on the abundance of photoautotrophic OTUs (*envfit* results).
Please note: Wiley is not responsible for the content or functionality of any Supporting Information supplied by the authors. Any queries (other than missing material) should be directed to the *New Phytologist* Central Office.

## Data Availability

The data that support the findings of this study are openly available in IDA (ida.fairdata.fi) research data storage service (Kuuri‐Riutta *et al*., 2025, doi: 10.23729/fd‐bad048dd‐eda3‐380f‐915a‐580ccd150ec2); the sequence data are stored in the NCBI Database with the accession no. PRJNA1307366. Moss photosynthesis data are stored in the IDA (Laine, 2022, doi: 10.23729/b6e83383‐8720‐406e‐8fd0‐4a2094ade983); the moss coverage data used to calculate the *P*_max_ are stored in the Dryad repository (Köster *et al*., 2024, doi: 10.5061/dryad.dz08kps39); the environmental data from Kokkonen *et al*. (2019a) are stored in the Pangaea Data Library (doi: 10.1594/pangaea.904256).
