# Supplementary material for: Microbial and bryospheric photosynthesis of boreal peatlands have peatland‐type‐specific responses to long‐term drying
Source: New Phytol. 2025 Sep 2;248(3):1336–50. doi: 10.1111/nph.70519 (PMC12489279; doi:10.1111/nph.70519)
Supplement: Supplementary file 1 — Fig. S1 Rarefaction curves in each study area. Fig. S2 Diversity indices from photoautotrophic microbial communities. Fig. S3 Correlations between the photosynthetic parameters and variables that best explained them according to the comparison of linear models (see Table 2, for statistical details.). Table S1 Summary of the measured environmental variables. Table S2 Statistical comparison of environmental variables, decomposition parameters, photosynthetic parameters, diversity indices, and the absolute abundance of the dominant photoautotrophic phyla between the water level treatments and the three different study sites. Table S3 Univariate test results on the impact of water level drawdown, site, and their interaction on the most abundant (relative abundance ≥ 0.01%) photoautotrophic phyla and orders. Table S4 Univariate test results on the impact of water level drawdown, site, and their interaction on the most abundant (relative abundance ≥ 0.01%) photoautotrophic genera. Table S5 Model diagnostic statistics for structural equation models. Table S6 Results of the multivariate generalised linear model on the impact of water level drawdown, site, and their interaction on the different taxonomic levels of the photoautotrophic community. Table S7 Explaining power of different taxonomic groups in the non‐metric multidimensional scaling based on the abundance of photoautotrophic OTUs (envfit results). Please note: Wiley is not responsible for the content or functionality of any Supporting Information supplied by the authors. Any queries (other than missing material) should be directed to the New Phytologist Central Office. [file NPH-248-1336-s001.pdf]

New Phytologist Supporting Information

Article title: Microbial and bryospheric photosynthesis of boreal peatlands have peatland-type-specific responses to long-term drying
Authors: Kuuri-Riutta, O., Le Geay, M., Jassey, V.E.J., Barel, J.M., Laine, A.M., Ylänne, H., Tuittila E.-S
Article acceptance date: 04 August 2025

The following Supporting Information is available for this article:

Table S1. The summary of the measured environmental variables.

Fig. S1. Rarefaction curves in each study area.

Table S2. Statistical comparison (two-way-ANOVA and the non-parametric equivalent Scheirer-Ray-Hare) of environmental variables, decomposition parameters, photosynthetic parameters, diversity indices, and the absolute abundance of the dominant photoautotrophic phyla between the water level treatments (control and WLD) and the three different study sites.

Table S3. Univariate test results on the impact of water level drawdown (Treatment), Site (rich fen, poor fen and bog) and their interaction on the most abundant (relative abundance ≥ 0.01 %) photoautotrophic phyla and orders.

Table S4. Univariate test results on the impact of water level drawdown (Treatment), Site (rich fen, poor fen and bog) and their interaction on the most abundant (relative abundance ≥ 0.01 %) photoautotrophic genera.

Table S5. Model diagnostic statistics for structural equation models.

Table S6. Results of the multivariate generalised linear model on the impact of water level drawdown (Treatment), Site (rich fen, poor fen and bog) and their interaction on the different taxonomic levels of the photoautotrophic community.

Fig. S2. Diversity indices from photoautotrophic microbial communities.

Table S7. The explaining power of different taxonomic groups in the NMDS based on the abundance of photoautotrophic OTU's (*envfit* results).

Fig. S3. Correlations between the photosynthetic parameters and variables that best explained them according to the comparison of linear models (See Table 2 for statistical details.)

Table S1. The summary of the measured environmental variables.

**Variable**	**Abbreviation**	**Unit**	**Measurement year**	**Repetitions**	**Description or reference**
Soil nutrient concentrations (Ca, Fe, K, N, P, Mg, S)		g^{-1} kg^{-1}	2016	1	Kokkonen *et al*. (2019a, b)
Water table level	WT	cm	2022	Every 2nd week, Jun-Aug	A distance from peat surface to water level, measured from permanent water wells. Negative value means below peat surface.
soil pH			2016	1	Kokkonen *et al*. (2019a, b)
Shading intensity	Shade	% of the initial PAR	2022	1	Quantifies the shading intensity from vascular plant coverage. 100–(PAR_{below}/PAR_{above}), where PAR_{below} is photosynthetically active radiation below the field layer and PAR_{above} is photosynthetically active radiation above field layer.
moss water content	Moss moisture	% of weigh	2022	1	100–(moss dry mass / moss fresh mass)
Soil temperature at 5 and 15 cm depth	SoilT5, SoilT15	°C	2022	Every 2nd week Jun-Aug	Measured from the permanent sampling points.

Fig. S1. Rarefaction curves in each study area.

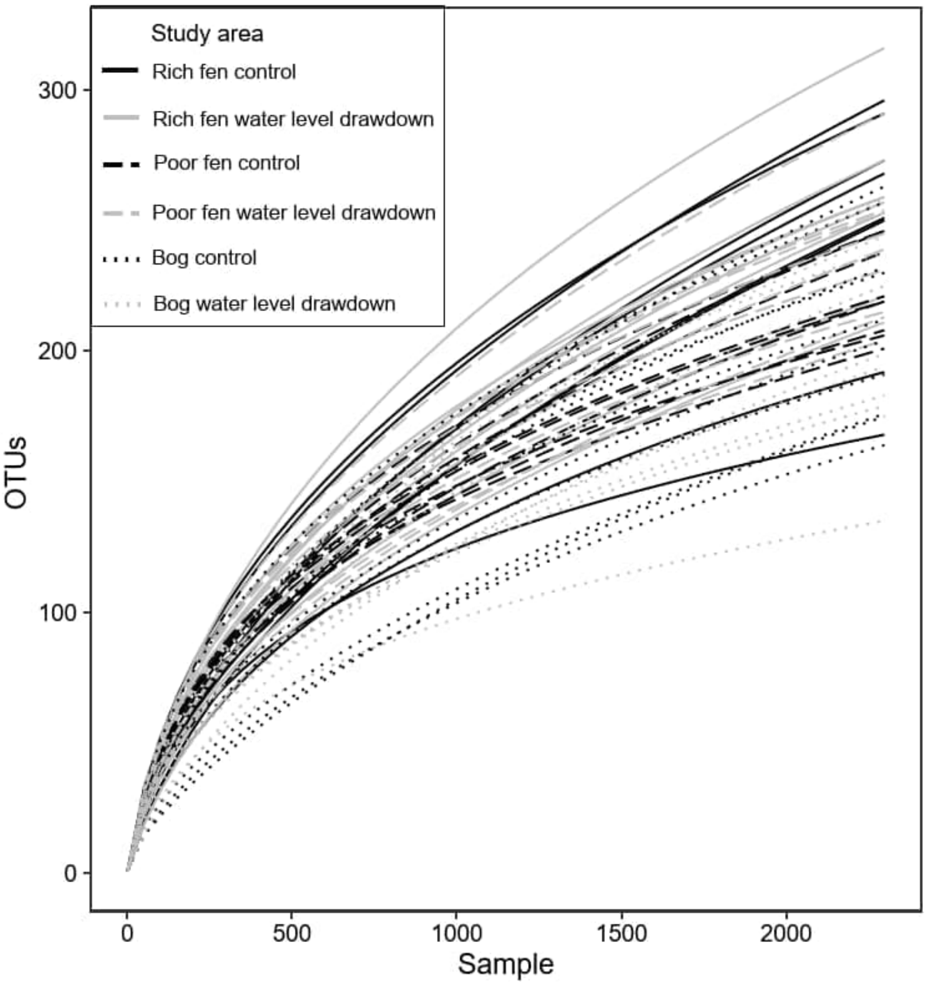

Table S2. Statistical comparison of environmental variables, decomposition parameters, photosynthetic parameters, diversity indices, and the absolute abundance of the dominant photoautotrophic phyla between the water level treatments (control and WLD) and the three different study sites (rich fen, poor fen, bog). The tests used were two-way-ANOVA and non-parametric equivalent Scheirer-Ray-Hare (indicated with *). For the significant site or treatment effects and interactions, significant pair-wise differences ($p \leq 0.05$) as identified by Tukey's post hoc test are marked with letters. For the variables inspected with the non-parametric test, the interpretation is based on visual inspection of box plots.

	**Treatment**		**Site**		**Site:Treatment**	
	F or *H	p-value	F or *H	p-value	F or *H	p-value
Water table	37.93	0.000 a	8.80	0.000 c	0.75	0.48
Moss moisture content	16.61	0.000 a	10.22	0.000 c	0.25	0.781
pH	4.84	0.033 a	77.53	0.000 d	7.9	0.001 j
Soil temperature at 5 cm depth	31.63	0.000 i	6.34	0.004 a	2.83	0.07
Shading intensity	5.62*	0.018 b	30.39*	0.000 d	7.63*	0.022 k
Nitrogen concentration	6.64	0.013 b	47.02	0.000 d	3.86	0.028 k
Phoshorus concentration	20.64	0.000 b	102.66	0.000 d	10.07	0.000 k
Sulphur concentration	2.35	0.132	31.123	0.000 d	6.44	0.003 k
Iron concentration	0.35*	0.552	30.24*	0.000	0.54*	0.762
Magnesium concentration	0.72	0.4	57.74	0.000 d	3.06	0.056
Potassium concentration	15.46	0.000 a	3.50	0.038 h	2.71	0.077
Mn	0.89	0.351	18.36	0.000 e	0.53	0.594
Aluminium concentration	0.74	0.393	1.35	0.27	0.28	0.759
Calsium concentration	2.13	0.152	132.47	0.000 d	1.84	0.17
Decomposition rate (*k*TBI)	8.415	0.006 b	0.98	0.384	0.60	0.552
Electron transport rate (ETR_{MAX})	2.9	0.095	19.95	0.000 f	5.56	0.001 k
Quantum yield of photosystem II (ϕPSII)	5.6	0.022 b	6.37	0.004 g	1.22	0.304
Bryospheric photosynthesis (P_{MAX})	35.77	0.000 b	0.92	0.4	20.56	0.000 k
Shannon index	0.1	0.754	7.44	0.002 h	0.41	0.665
Simpson's index	0.99	0.326	8.77	0.001 e	0.40	0.675
OTU richness	0.43	0.514	7.52	0.001 i	0.42	0.663
Hill's evenness	0.65	0.424	11.11	0.000 e	0.84	0.438
Absolute abundance of all photoautotrophic microbes	14.71	0.000 b	5.48	0.007	3.4	0.042 k
Absolute abundance of Chlorophyta	7.12*	0.008 b	16.31*	0.000 e	0.53*	0.766

Absolute abundance of cyanobacteria	4.42	0.041 b	11.26	0.000 c	3.38	0.043 k
Absolute abundance of Glaucophyta	1.66	0.204	1.59	0.215	2.05	0.141
Absolute abundance of Ochrophyta	0.2*	0.658	0.56*	0.755	1.44*	0.487
Absolute abundance of Streptophyta	1.75	0.186	0.02	0.898	0.29	0.752

a. higher in control areas

b. higher in water level drawdown areas

c. lower in the poor fen than in the other sites

d. all sites differ from each other; highest in the rich fen, intermediate in the poor fen and lowest in the bog

e. higher in the poor fen than in the other sites

f. higher in the rich fen than in the other sites

g. higher in the rich fen and poor fen than in the bog (poor and rich fen did not differ from each other)

h. higher in poor fen than bog

i. higher in the rich fen than in the bog (poor fen intermediate but no significant difference between rich fen – poor fen or poor fen – bog).

j. Significant difference between treatments only in the rich fen

k. Significant difference between treatments only in the poor fen

Table S3. Univariate test results on the impact of water level drawdown (treatment), site (rich fen, poor fen and bog), and their interaction on the absolute abundance of the most abundant (relative abundance (rel. abu) ≥ 0.01 %) photoautotrophic phyla and orders (from *mvabund*; see the results for the overall multivariate test in Table S6). If a phylum consisted of only one order, statistics are shown only for the phylum level and the name of the order is defined in parentheses after phylum name. Unadjusted p-value is shown as a number (significant in bold), and the significance of adjusted p-value is indicated with asterisks (* = p ≤ 0.05, ** = p ≤ 0.01, *** = p ≤ 0.001). WLD = water level drawdown, CON = control, RF = rich fen, PF = poor fen, BO = bog.

**Phylum / (Order)**	**Rel. Abu (%)**	**Treatment**		**Site**		**Site*Treatment**		**Preferred treatment**	**Preferred site**
		**Dev.**	**p**	**Dev.**	**p**	**Dev.**	**p**		
Cercozoa (Chlorarachnida)	0.01	1.24	0.214	7.23	**0.026**	0.01	0.815		
Chlorophyta	38.03	11.54	**0.004***	21.75	**0.001****	0.73	0.746	WLD	PF
Chlamydomonadales	6.14	3.43	0.145	6.77	0.158	8.38	0.064		
Chlorellales	4.4	3.68	0.122	30.15	**0.001*****	0.92	0.747		PF
Chlorophyceae	0.01	5.25	0.061	7.83	**0.014**	0.00	0.344		RF
Mamiellales	0.02	0.73	0.343	0.42	0.706	3.06	0.445		
Microthamniales	0.18	6.53	**0.001**	0.25	0.697	0.06	0.929	WLD	
Pedinomonadales	0.02	0.36	0.591	3.64	0.243	6.25	**0.039**		
Prasinococcales	19.84	11.64	**0.003**	32.52	**0.001*****	0.04	0.984	WLD	PF, RF
Prasiolales	4.04	3.76	0.093	9.14	**0.045**	11.10	**0.027**	WLD in RF, CON in PF	PF
Sphaeropleales	0.28	4.75	**0.017**	12.25	**0.003**	5.98	0.056	CON	RF, PF
Trebouxiophyceae	0.48	1.64	0.212	4.72	0.455	4.74	0.093		
Ulotrichales	0.01	0.12	0.7	3.91	0.177	6.29	**0.023**		
Watanabea Clade	0.06	3.79	**0.025**	15.89	**0.002***	8.24	**0.016**	WLD	RF, PF
Cryptophyta (Cryptophyceae)	0.41	0.62	0.368	2.27	0.219	0.76	0.621		
Cyanobacteria	39.12	5.73	**0.04**	13.25	**0.004***	18.76	**0.002****	WLD in PF	RF, BO
Eurycoccales	0.32	3.10	0.051	20.20	**0.001***	5.34	0.072		RF
Gloeobacterales	0.01	0.21	0.629	3.94	0.199	1.85	0.328		BO
Leptolyngbyales	0.34	4.72	**0.033**	23.25	**0.001****	5.07	0.058	CON	RF
Nostocales	38.16	6.34	**0.037**	12.46	**0.003**	18.07	**0.006***	WLD in PF	
Synechococcales	0.08	3.11	**0.008**	3.03	**0.038**	4.84	**0.03**	WLD in PF & RF	RF & PF
Thermosynechococcales	0.01	0.13	0.642	0.06	0.895	6.81	0.077		
Dinoflagellata (Gymnodiniales)	0.04	2.20	**0.027**	3.52	**0.024**	1.71	0.261	WLD	PF

Discoba (Eugledina)	1.4	3.91	**0.016**	1.29	0.534	1.83	0.282	WLD	RF & PF
Glaucophyta (Glaucocystophytes)	0.08	0.05	0.824	4.36	0.164	3.01	0.266		
Ochrophyta	18.38	0.01	0.93	1.67	0.539	5.22	0.148		
Bacillariophyta	8.19	0.68	0.451	13.19	**0.008**	2.75	0.339		RF
Chromulinales	1	3.96	**0.005**	3.16	0.052	2.49	0.081	CON	
Chrysophyceae	0.34	1.78	0.071	1.21	0.57	1.92	0.268		
Eustigmatophyceae	6.29	1.58	0.311	2.84	0.325	14.58	**0.003**	CON in RF, WLD in PF & BO	
Sarcinochrysidales	0.01	6.06	**0.022**	3.10	0.289	0.00	0.773	CON	
Synurales	2.52	6.39	**0.003**	0.06	0.967	7.30	**0.011**	CON	
Xanthophyceae	0.01	0.27	0.561	0.68	0.431	5.42	0.142		
Rhodophyta	0.3	1.13	0.218	15.06	**0.001***	8.37	**0.035**	RF	
Bangiales	0.28	1.66	0.246	20.87	**0.001****	4.35	0.135	RF	
Cyanidiales	0.01	3.51	0.058	0.07	0.897	3.33	0.177		
Streptophyta	2.23	2.43	0.147	6.05	0.327	4.78	0.13		
Klebsormidiophyceae	0.23	1.61	0.081	0.17	0.857	1.30	0.301		
Mesostigmatophyceae	0.05	0.09	0.717	24.44	**0.001*****	0.00	0.692	BO	
Zygnemophyceae	1.95	3.60	0.07	5.46	0.396	4.30	0.14		

Table S4. Univariate test results on the impact of water level drawdown (treatment), site (rich fen, poor fen and bog), and their interaction on the absolute abundance of the most abundant (relative abundance (rel. abu) ≥ 0.01 %) photoautotrophic genera (from *mvabund*; see the results for the overall multivariate test in Table S6). Unadjusted p-value is shown as a number (significant in bold), and the significance of adjusted p-value is indicated with asterisks (* = p ≤ 0.05, ** = p ≤ 0.01, *** = p ≤ 0.001). WLD = water level drawdown, CON = control, RF = rich fen, PF = poor fen, BO = bog.

Phylum / Order	Genus	Rel Abu (%)	Treatment		Site		Site*Treatment		Preferred treatment	Preferred site
			Dev.	p	Dev.	p	Dev.	p		
Cercozoa / Chlorarachnida	*Gymnochlora*	0.01	1.24	0.164	7.23	**0.032**	0.01	0.857		PF
Chlorophyta/ Chlamydomonadales	*Carteria*	0.09	2.72	**0.041**	1.79	0.12	6.78	0.088	CON	
	Characiochloris	0.01	2.34	0.153	0.58	0.623	8.71	**0.03**		
	Chlamydomonas	1.2	3.97	**0.041**	14.83	**0.001**	4	0.096	WLD	PF, RF
	Chloromonas	2.14	2.79	0.16	11.68	0.086	5.29	0.185		
	Coccomyxa	1.43	8.77	**0.003**	9.85	**0.005**	6.11	0.064	WLD	PF, RF
	Ettlia	1.06	0.7	0.183	10.41	**0.001**	1.09	0.277		BO
	Ignatius	0.02	0.27	0.627	1.27	0.316	6.24	0.113		
	Pleurastrum	0.02	1.16	0.271	1.25	0.302	2.7	0.453		
	Volvox	0.06	8.72	**0.013**	11.6	**0.006**	0	0.378	CON	BO
Chlorophyta / Chlorellales	*Chlorella*	3.81	0.49	0.509	29.4	**0.001*****	3.33	0.272		PF
	Lobosphaera	0.13	4.74	**0.003**	2.62	0.12	3.62	**0.041**	WLD in RF & PF	
	Neglectella	0.28	1.57	0.1	3.5	**0.037**	1.65	0.365		BO
	Neocystis	0.14	1.57	0.096	5.78	**0.013**	2.17	0.213		RF
Chlorophyta / Chlorophyceae	*Treubaria*	0.01	5.25	**0.025**	7.83	**0.013**	0	0.393	WLD	RF
Chlorophyta / Mamiellales	*Bathycoccus*	0.02	0.73	0.344	0.42	0.68	3.06	0.433		
Chlorophyta / Microthamniales	*Myrmecia*	0.06	0.35	0.399	2.03	0.124	0.53	0.661		
	Trebouxia	0.11	7.18	**0.003**	3.46	**0.016**	0.24	0.82	WLD	RF & PF
Chlorophyta / Pedinomonadales	*Pedinomonas*	0.02	0.36	0.597	3.64	0.233	6.25	**0.041**	CON in RF	

Chlorophyta Prasinococcales	*Prasinoderma*	19.84	11.64	**0.001**	32.52	**0.001*** **	0.04	0.98	WLD	PF & RF
Chlorophyta / Prasiolales	*Elliptochloris*	3.24	2.15	0.214	8.97	**0.04**	10.96	**0.019**	WLD in RF	
	Koliella	0.01	0.88	0.367	3.85	0.196	1.96	0.334		
	Prasiolopsis	0.04	6.85	**0.006**	2.81	0.091	4.32	0.119	WLD	
Chlorophyta / Sphaeropleales	*Tetradesmus*	0.27	5.71	**0.019**	10.85	**0.007**	4.62	0.129	CON	RF
Chlorophyta / Trebouxiophyceae	*Botryococcus*	0.04	2.47	0.113	2.38	0.171	13.63	**0.004**	WLD in RF	
	Microthamnion	0.04	0.52	0.343	16.79	**0.001***	0.1	0.723		BO
	Xylochloris	0.4	1.27	0.134	1.75	0.526	1.58	0.31		
Chlorophyta / Watanabea-Clade	*Watanabea*	0.06	3.79	**0.013**	15.89	**0.001***	8.24	**0.018**	WLD	RF & PF
Cryptophyta / Cryptophyceae	*Cryptomonas*	0.41	0.62	0.379	2.27	0.222	0.76	0.603		
Cyanobacteria / Eurycoccales	*Synechococcus*	0.32	3.1	**0.033**	20.2	**0.005***	5.34	0.053	CON	RF
Cyanobacteria / Leptolyngbyales	*Leptolyngbya*	0.34	4.72	**0.045**	23.25	**0.001****	5.07	0.052	CON	RF
Cyanobacteria Nostocales	*Anabaena*	8.04	0.08	0.741	25.05	**0.001****	1.36	0.432		BO
	Calothrix	0.02	0.1	0.654	0.56	0.408	4.83	0.216		
	Chroococcidiopsis	5.63	21.61	**0.001****	40.39	**0.001*****	3.32	0.225	WLD	PF, RF
	Crinalium	0.03	0.8	0.163	0.19	0.712	0.12	0.854		
	Cyanothece	0.13	0.71	0.295	5.94	**0.002**	2.31	0.21		RF
	Cylindrospermum	8.59	0.33	0.449	2.68	**0.046**	2.38	0.147		RF
	Dolichospermum	1.26	1.72	0.192	20.81	**0.001***	2.65	0.197		BO
	Gloeocapsa	0.03	7.75	**0.003**	10.8	**0.005**	0	0.161	CON	RF
	Mastigocladopsis	0.03	1.08	0.1	1.02	0.209	0.64	0.525		
	Microcoleus	0.77	5.35	0.065	13.13	**0.002**	1.02	0.339		RF
	Nostoc	8.45	6.74	**0.02**	4.51	0.247	9.87	**0.014**	WLD in RF	
	Pleurocapsa	0.02	0.21	0.706	10.85	**0.004**	0	0.745		RF
	Prochloron	0.01	2.98	0.141	1.89	0.587	0	0.596		
Cyanobacteria / Oxyphotobacteria Inc. Sed.	*Pseudanabaena*	0.12	1.56	0.113	17.59	**0.002***	0.02	0.892		BO & RF
Cyanobacteria / Synechococcales	*Prochlorothrix*	0.08	3.18	**0.021**	3.18	**0.038**	4.04	**0.036**	WLD in RF & PF	RF & PF
Cyanobacteria / Thermosynechococcales	*Thermosynechococcus*	0.01	0.13	0.671	0.06	0.903	6.81	0.057		
Dinoflagellata / Gymnodiniales	*Karlodinium*	0.04	4.48	**0.011**	4.85	**0.01**	0.41	0.744	WLD	RF & PF
Discoba / Euglenida	*Cryptoglena*	0.07	0.03	0.761	0.25	0.729	0.28	0.75		
	Euglena	1.31	3.36	**0.026**	1.09	0.537	1.57	0.341	WLD	
	Trachelomonas	0.01	0.59	0.315	7.75	**0.031**	6	**0.048**	CON in PF	PF & BO
Glaucophyta / Glaucocystophytes	*Glaucocystis*	0.08	0.05	0.807	4.36	0.176	3.01	0.271		
Ochrophyta / Bacillariophyta	*Eunotia*	2.74	0.12	0.69	6.42	**0.026**	0.25	0.844		RF
	Leptocylindrus	3.15	18.15	**0.001****	14.88	**0.002**	1.68	0.461	WLD	PF
	Nitzschia	0.42	13.48	**0.001***	0.4	0.837	0.15	0.915	WLD	

	Phaeodactylum	0.82	5.83	**0.02**	22.58	**0.001****	3.16	0.143	CON	RF
	Thalassiosira	0.01	0.08	0.744	0.45	0.598	2.66	0.553		
Ochrophyta / Chromulinales	*Chromulina*	1	3.96	**0.006**	3.16	0.061	2.49	0.084	CON	
Ochrophyta / Chrysophyceae	*Ochromonas*	0.25	2.31	**0.025**	0.92	0.63	13.35	**0.006**	CON	
	Poterioochromonas	0.08	0.19	0.468	0.18	0.768	0.04	0.96		
Ochrophyta / Eustigmatophyceae	*Nannochloropsis*	6.28	1.54	0.277	2.83	0.328	14.27	**0.002**	WLD in PF & BO, CON in RF	
Ochrophyta / Sarcinochrysidales	*Aureoumbra*	0.01	6.06	**0.034**	3.1	0.298	0	0.525	CON	
Ochrophyta / Synurales	*Mallomonas*	1.74	5.56	**0.005**	0.33	0.786	7.55	**0.011**	CON	
	Synura	0.59	3.17	**0.042**	2.7	0.224	5.89	**0.033**	CON in PF & in BO	
Ochrophyta / Xanthophyceae	*Vaucheria*	0.01	0.27	0.553	0.68	0.438	5.42	0.137		
Rhodophyta / Bangiales	*Porphyra*	0.28	0	0.987	34.47	**0.001*****	0	0.711		RF
Streptophyta / Klebsormidiophyceae	*Klebsormidium*	0.23	1.61	0.073	0.17	0.878	1.3	0.315		
Streptophyta / Mesostigmatophyceae	*Chaetosphaeridium*	0.05	0.09	0.759	24.44	**0.001****	0	0.744		BO
Streptophyta / Zygnemophyceae	*Actinotaenium*	0.04	0.02	0.894	7.26	**0.035**	0.73	0.558		RF
	Cylindrocystis	0.12	1.29	0.19	0.73	0.604	3.41	0.356		
	Fottea	0.34	2.11	0.061	1.36	0.342	4.67	0.064		
	Mesotaenium	0.24	4.15	**0.005**	3.3	**0.009**	17.06	**0.002**	WLD	PF & RF
	Mougeotia	0.73	1.25	0.339	3.79	0.271	6.35	**0.049**	CON in BO	
	Netrium	0.13	0.64	0.227	2.31	0.206	0.34	0.717		
	Penium	0.11	2.94	0.075	3.64	0.057	7.39	0.077		

Table S5. Model diagnostic statistics for structural equation models. Models were built separately for control and WLD (water level drawdown areas). Diagnostic statistics are shown for the first tested models (priori) and finally selected models (final).

Model	Fisher's c	$p_{Fisher's\ c}$	Chi-squared	$p_{Chi\text{-}squared}$
$Priori_{control}$	17.46	0.23	12.49	0.085
$Final_{control}$	6.93	0.86	3.15	0.79
$Priori_{WLD}$	17.78	0.22	11.73	0.11
$Final_{WLD}$	9.12	0.69	4.24	0.64

Table S6. Results of the overall multivariate generalised linear model (*mvabund*) on the impact of water level drawdown (treatment), site (rich fen, poor fen and bog) and their interaction on the absolute abundance of phyla, orders, and genera of the photoautotrophic microbial community. Unadjusted p-value is shown as a number, and the significance of adjusted p-value is indicated with asterisks. (* = $p \leq 0.05$, ** = $p \leq 0.01$, *** = $p \leq 0.001$). The associated univariate results for the absolute abundance of photoautotrophic phyla and orders are found in Table S3 and for genera in Table S4.

	Treatment		Site		Site*Treatment	
	**Dev.**	**p**	**Dev.**	**p**	**Dev.**	**p**
Phylum	30.35	**0.002****	78.93	**0.001*****	45.19	**0.01***
Order	127.89	**0.001*****	322.08	**0.001*****	166.36	**0.001*****
Genus	296.19	**0.001*****	733.30	**0.001*****	290.36	**0.002*****

Fig. S2. Diversity indices from photoautotrophic microbial communities. The differences were significant between the sites (p-values < 0.05), but not between the treatments (p-values >0.05), nor was there a significant interaction (p-values >0.05). The violins illustrate the distribution of the data and the kernel probability density of the data at different values. The yellow dots indicate mean values, and the whiskers show standard deviation. Letters indicate significant differences according to Tukey's pairwise comparison (p-value <0.05).

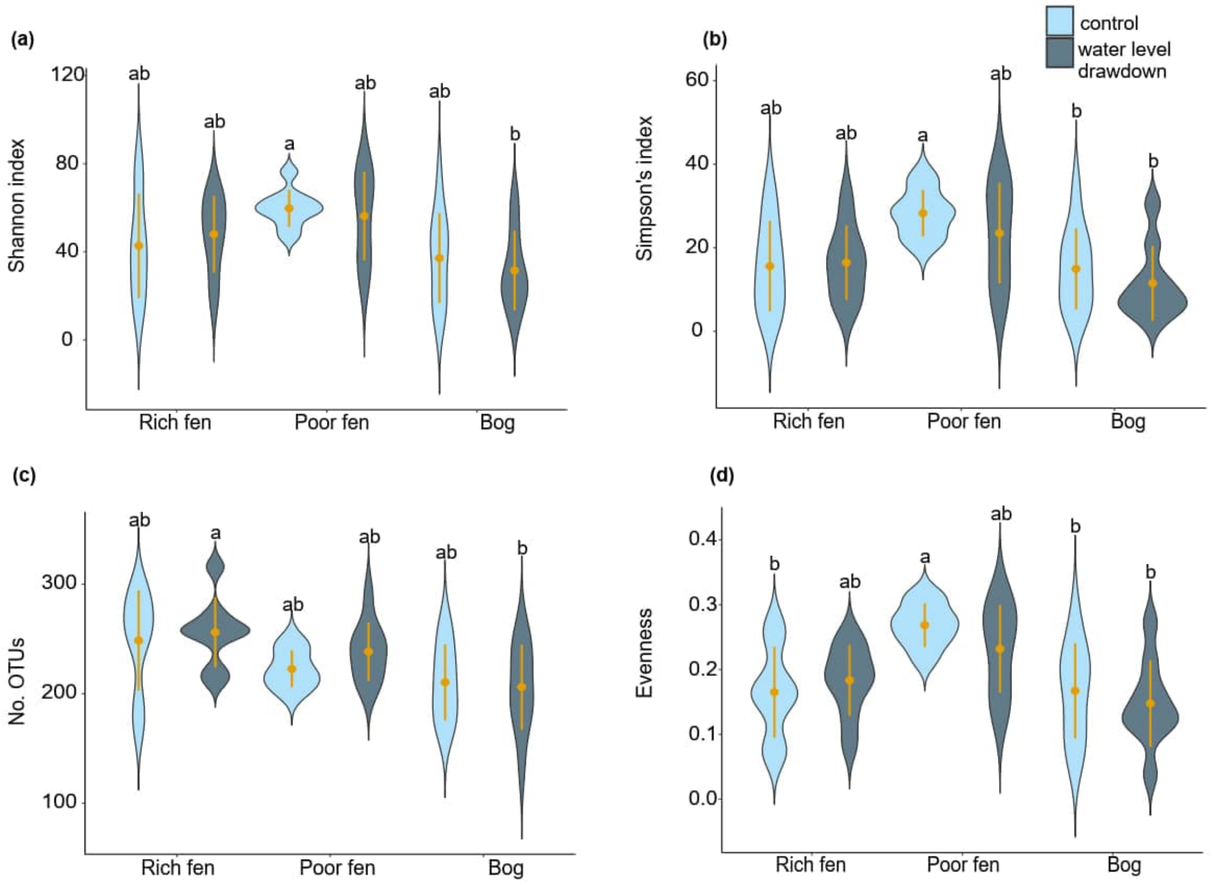

Table S7. The explaining power of different taxonomic groups in the NMDS analyses based on the absolute abundance of photoautotrophic OTU's (*envfit* results; Fig 5).

	r^2	Pr(>r)
**Phyla**		
Chlorophyta	0.43	0.001
Cyanobacteria	0.19	0.006
Discoba	0.01	0.851
Ochrophyta	0.08	0.119
Streptophyta	0.33	0.002
**Cyanobacteria families**		
Chroococcidiopsaceae	0.32	0.001
Coleofasciculaceae	0.10	0.100
Eurycoccales	0.16	0.016
Gloeobacteraceae	0.20	0.011
Leptolyngbyaceae	0.36	0.001
Microcystaceae	0.13	0.033
Nostocaceae	0.21	0.002
Prochlorotrichaceae	0.19	0.012
Thermosynechococcaceae	0.03	0.511
**Chlorophyta orders**		
Chlamydomonadales	0.16	0.014
Chlorellales	0.14	0.025
Chlorophyceae	0.04	0.318
Microthamniales	0.08	0.106
Prasinococcales	0.24	0.001
Prasiolales	0.33	0.001
Sphaeropleales	0.08	0.102
Trebouxiophyceae	0.13	0.058
Watanabea-Clade	0.33	0.001
**Chlorophyta orders**		
Bacillariophyta	0.28	0.001
Chromulinales	0.22	0.002
Eustigmatophyceae	0.10	0.087
Synurales	0.13	0.030
Xanthophyceae	0.07	0.164

Fig. S3. Correlations between the photosynthetic parameters and variables that best explained them according to the comparison of linear models (Table 2). Rho and p-values are from Spearman correlations. ETR_{max} = electron transport rate, ΦPSII = quantum yield of photosystem II, P = phosphorus concentration, Chlorophyta = the absolute abundance of phylum Chlorophyta, Cyanobacteria = the absolute abundance of phylum Cyanobacteria, PC1 = PC1 axis from PCA based on environmental variables (representing the effect of site fertility and pH gradient) S = sulphur concentration, k_{TBI} = decomposition rate.

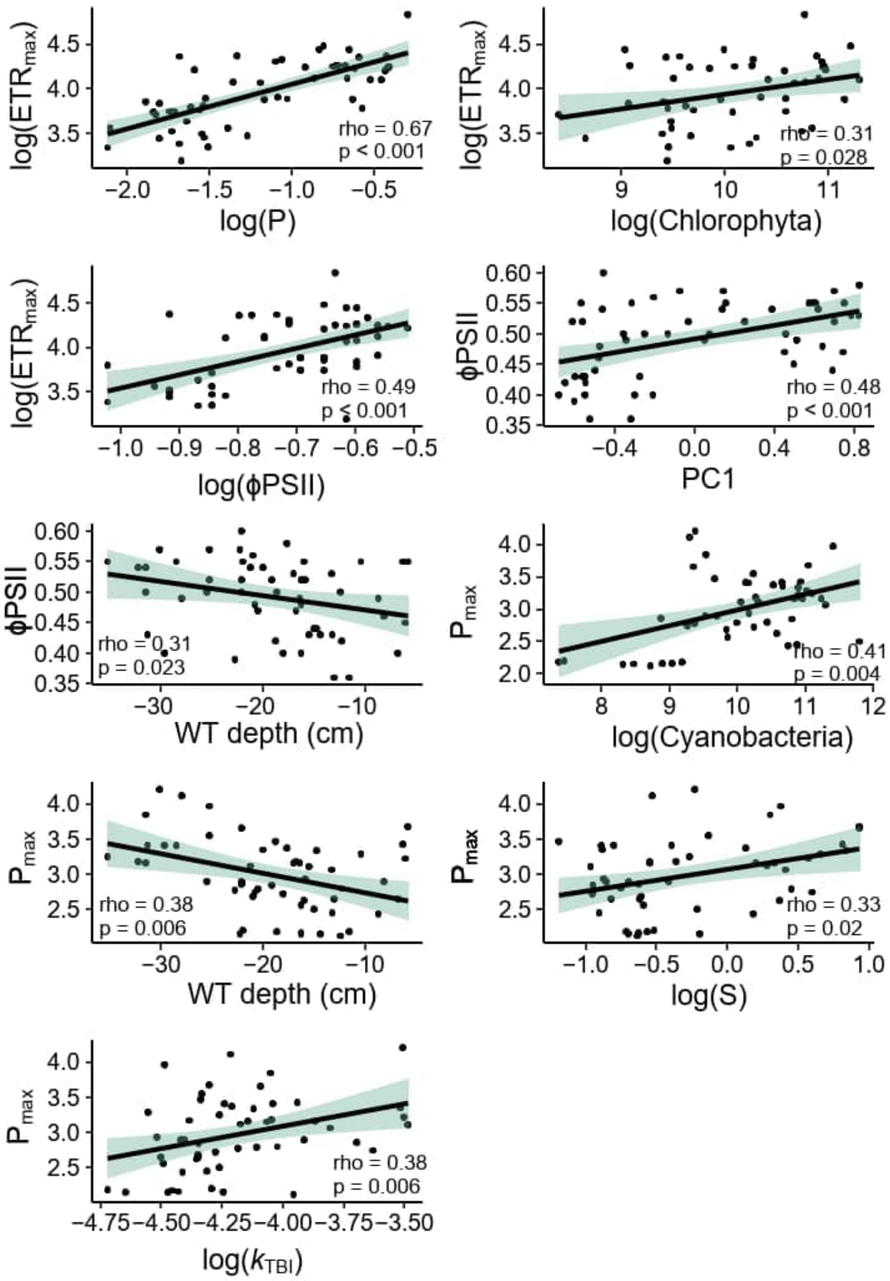

log(ETRmax)
log(P)
rho = 0.67
p < 0.001
log(Chlorophyta)
rho = 0.31
p = 0.028
log(ϕPSII)
rho = 0.49
p < 0.001
ϕPSII
PC1
rho = 0.48
p < 0.001
WT depth (cm)
rho = 0.31
p = 0.023
Pmax
log(Cyanobacteria)
rho = 0.41
p = 0.004
rho = 0.38
p = 0.006
log(S)
rho = 0.33
p = 0.02
log(kTBI)
rho = 0.38
p = 0.006

References

Kokkonen NAK, Laine AM, Laine J, Vasander H, Kurki K, Gong J, Tuittila E-S, Collins B. 2019a. Responses of peatland vegetation to 15-year water level drawdown as mediated by fertility level. *Journal of vegetation science* 30(6): 1206-1216. https://doi.org/10.1111/jvs.12794

Kokkonen NAK, Laine AM, Laine J, Vasander H, Kurki K, Gong J., Tuittila E.-S. 2019b. Data for: *Responses of peatland vegetation to 15-year water level drawdown as mediated by fertility level*. PANGAEA Data Publisher for Earth & Environmental Science. https://doi.org/10.1594/PANGAEA.904256
